# BCR-ABL triggers a glucose-dependent survival program during leukemogenesis through the suppression of TXNIP

**DOI:** 10.1038/s41419-023-05811-2

**Published:** 2023-04-24

**Authors:** Lin Feng, Ruxin Ding, Xuan Qu, Yuanchun Li, Tong Shen, Lei Wang, Ruikai Li, Juan Zhang, Yi Ru, Xin Bu, Yang Wang, Min Li, Wenqi Song, Liangliang Shen, Pengxia Zhang

**Affiliations:** 1 Key Laboratory of Microecology-immune Regulatory Network and Related Diseases, School of Basic Medicine, Jiamusi University, Jiamusi, Heilongjiang China; 2 Shaanxi University of Chinese Medicine, Xianyang, China; 3 The State Key Laboratory of Cancer Biology, Department of Biochemistry and Molecular Biology, Fourth Military Medical University, Xi’an, China; 4 Department of Hematology, Tangdu Hospital, Fourth Military Medical University, Xi’an, China; 5 Department of Digestive Surgery, Xi’an International Medical Center, Xi’an, China; 6 Xi’an Beihuan Hospital, Xi’an, China; 7 Department of Gastrointestinal Surgery, Xijing Hospital, Fourth Military Medical University, Xi’an, China; 8 Department of Biochemistry and Molecular Biology, College of Life Sciences, Northwest University, Xi’an, China; 9 Tongchuan People’s Hospital, Tongchuan, China; 10 Xi’an Eastern Hospital, Xi’an, China; 11 Jiamusi Maternal and Child Health Care Hospital, Jiamusi, Heilongjiang China

**Keywords:** Cancer metabolism, Transcription, Chronic lymphocytic leukaemia

## Abstract

Imatinib is highly effective in the treatment of chronic myelogenous leukemia (CML), but the primary and acquired imatinib resistance remains the big hurdle. Molecular mechanisms for CML resistance to tyrosine kinase inhibitors, beyond point mutations in BCR-ABL kinase domain, still need to be addressed. Here, we demonstrated that thioredoxin-interacting protein (TXNIP) is a novel BCR-ABL target gene. Suppression of TXNIP was responsible for BCR-ABL triggered glucose metabolic reprogramming and mitochondrial homeostasis. Mechanistically, Miz-1/P300 complex transactivates TXNIP through the recognition of TXNIP core promoter region, responding to the c-Myc suppression by either imatinib or BCR-ABL knockdown. TXNIP restoration sensitizes CML cells to imatinib treatment and compromises imatinib resistant CML cell survival, predominantly through the blockage of both glycolysis and glucose oxidation which results in the mitochondrial dysfunction and ATP production. In particular, TXNIP suppresses expressions of the key glycolytic enzyme, hexokinase 2 (HK2), and lactate dehydrogenase A (LDHA), potentially through Fbw7-dependent c-Myc degradation. In accordance, BCR-ABL suppression of TXNIP provided a novel survival pathway for the transformation of mouse bone marrow cells. Knockout of TXNIP accelerated BCR-ABL transformation, whereas TXNIP overexpression suppressed this transformation. Combination of drug inducing TXNIP expression with imatinib synergistically kills CML cells from patients and further extends the survival of CML mice. Thus, the activation of TXNIP represents an effective strategy for CML treatment to overcome resistance.

## Introduction

Chronic myeloid leukemia (CML) is a lethal hematological malignancy resulting from a translocation between chromosome 9 and 22 that forms *bcr-abl* hybrid gene which occurs in over 90% of CML cases [1]. BCR-ABL activates multiple cell proliferation and survival pathways for hematopoietic stem cell (HSC) transformation [2]. Despite the success of BCR-ABL tyrosine kinase inhibitor (e.g., imatinib) results in complete cytogenetic response in most cases of chronic-phase CML, but results in poor responses in advanced phases of the disease, with frequent relapse [3]. Both primary and acquired resistance contribute to recurrent disease. Although point mutations in the ABL kinase domain appear to be the main cause of secondary resistance to imatinib, such point mutations do not appear to account for all of the resistance observed in patients with the accelerated form of CML [4–8]. Further understanding of the molecular mechanisms of the disease and resistance is critical for the development of effective therapeutic strategies in CML.

In CML, BCR-ABL activates glucose metabolism as part of its transforming activity. Activation of glycolysis in CML cells is associated with an increase in glucose transporter type 1 (GLUT-1) at the membrane and suppression of p53 [9–11]. In addition, BCR-ABL inhibitor imatinib reduced in surface localization of GLUT-1 and further decreased glucose uptake and lactate production. In particular, inhibition of glycolysis can enhance the imatinib cytotoxicity [10]. Thus glycolytic metabolism is suggested to play a key role in determining imatinib efficacy and provides a rationale for targeting glycolytic metabolism therapeutically [12, 13]. Moreover, targeting mitochondrial oxidative phosphorylation can eradicate therapy-resistant CML stem cells [14]. The combination of imatinib with oxidative phosphorylation inhibitor synergistically suppressed CML cells. Thus, identifying the key factors involved in the blockage of glucose metabolism, including both glycolysis and TCA cycle, may provide a new route for CML treatment.

Thioredoxin interacting protein (TXNIP) is a ubiquitous protein inhibitor of thioredoxin, an oxidoreductase that partners with thioredoxin reductase and thioredoxin peroxidase to reduce oxidized proteins and scavenge free radicals. So far, more and more evidence identified that TXNIP functions as a tumor suppressor *via* the inhibition of glucose uptake and aerobic glycolysis [15]. TXNIP is predominantly subjected to transcriptional regulation. When intracellular glucose levels are elevated, the transcription factor MondoA/MIx binds the E-box carbohydrate response element (ChoRE) in the TXNIP promoter, upregulating transcription as part of a negative feedback loop [16]. Conversely, MondoA/MIx activity is repressed when cells enter G1 and require biosynthetic glucose metabolites for anabolism [17].

Our group previously identified that TXNIP is a specific target of c-Myc. c-Myc represses its transcription by binding to ChoRE region, potentially competing with MondoA/Mlx complex [18]. Numerous studies further demonstrated the key roles of c-Myc/TXNIP axis in regulation of various cancer cell survival [19–21]. However, whether and how TXNIP regulates pathogenesis of CML was largely unknown. Furthermore, although TXNIP-mediated GLUT1 trafficking and internalization was identified [22], how TXNIP regulates a large scale of glycolytic enzyme expressions regrading to the inhibition of glucose metabolism still needs to be addressed. Here, we demonstrate that TXNIP expression was decreased in response to the activated BCR-ABL signaling, which is associated with a previously unappreciated mechanism that involves in c-Myc/Miz-1/P300 complex. Restoration of TXNIP expression sensitizes CML cells to imatinib treatment, potentially through the blockage of glucose metabolism. In particular, TXNIP suppressed glycolytic enzyme expressions through Fbw7-dependent c-Myc degradation. BCR-ABL suppression of TXNIP provided a novel survival pathway for CML transformation. Combination of drug activating TXNIP with imatinib effectively suppresses the development of CML-like myeloproliferative disease in mice, which provides a rationale for treating patients with CML with minimal residual disease.

## Results

### TXNIP is downregulated by BCR-ABL transformation

**Fig. 1:**
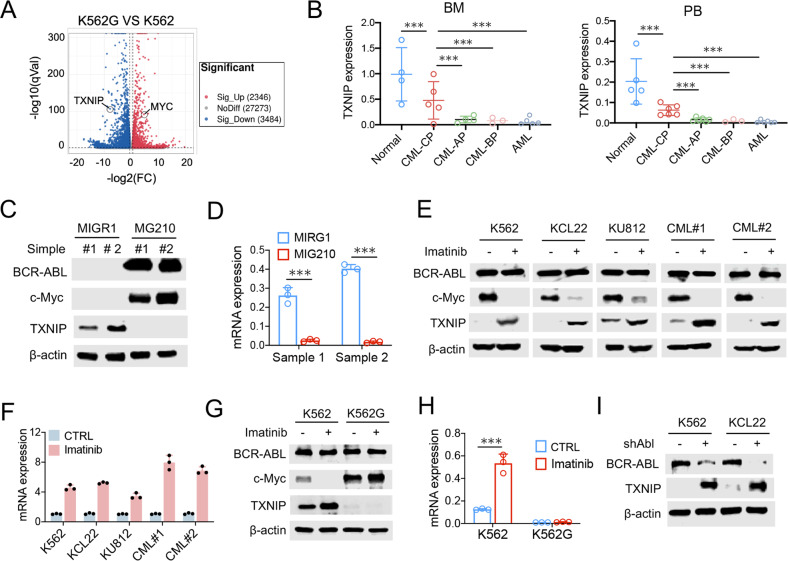
TXNIP is downregulated by BCR-ABL transformation. **A** Volcano plot representing the genes significantly differentially expressed in K562 and K562G (*P* < 0.05). **B** Relative mRNA levels of TXNIP in the PB and BM were compared among samples from normal healthy donors and patients as described in the figure. Each individual dot represents 1 sample. CP, chronic phase; AP, accelerated phase; BP, blast phase. **C**, **D** TXNIP protein (**C**) and mRNA (**D**) levels after BCR-ABL transduction (MIG210) in normal CD34^+^ cells from 2 independent healthy donors. Empty vector MIGR1 was used as a control. **E**, **F** TXNIP protein (**E**) and mRNA (**F**) levels in indicated CML cells 24 h after 0.5 μM imatinib treatment. **G**, **H** TXNIP protein (**G**) and mRNA (**H**) levels in K562 and K562G cells 24 h after 0.5 μM imatinib treatment. **I** TXNIP protein levels in K562 and KCL22 cells after BCR-ABL knockdown by shABL lentiviral vector. ***P < 0.001. **P < 0.01.

To determine the underlying mechanism of imatinib resistance in CML cells, we firstly generated imatinib-resistant K562/G01 (K562G) cells, and determined the gene expression profiles compared with the parental K562 cells by RNA sequencing assay (Fig. 1A). Intriguingly, TXNIP, which was verified the tumor suppressive role in several types of tumors in our previous studies [18, 21], was uncovered decreased in K562G cells (Fig. 1A and Supplementary Fig. 1). To determine whether TXNIP is associated with hematopoietic malignancies, we further analyzed TXNIP expression in the bone marrow (BM) and peripheral blood (PB) samples from normal healthy donors and patients with different types of myeloproliferative diseases, including CML and acute myeloid leukemia (AML). Compared with normal patients, TXNIP was dramatically downregulated in both the BM and PB of patients with chronic-phase CML (Fig. 1B).

Thus, this finding prompted the determination of the change of TXNIP expression during BCR-ABL transformation. Normal CD34^+^ cells were transduced with the BCR-ABL retroviral vector MIG210. The TXNIP protein was downregulated by BCR-ABL transduction along with TXNIP mRNA level (Fig. 1C, D). Moreover, inhibition of BCR/ABL by imatinib treatment increased both TXNIP protein and mRNA levels in CML cell lines (K562, KCL22, and KU812) and primary CML cells from patients (CML#1 and CML#2) (Fig. 1E, F). In contrast, the treatment did not change TXNIP expression in imatinib-resistant K562G cells (Fig. 1G, H). Similarly, when BCR-ABL was knocked down, TXNIP levels increased (Fig. 1I). These data further support that BCR-ABL suppresses TXNIP expression in CML cells.

### BCR-ABL blocks TXNIP transactivation by disrupting a Miz-1-p300 complex via c-Myc induction

**Fig. 2:**
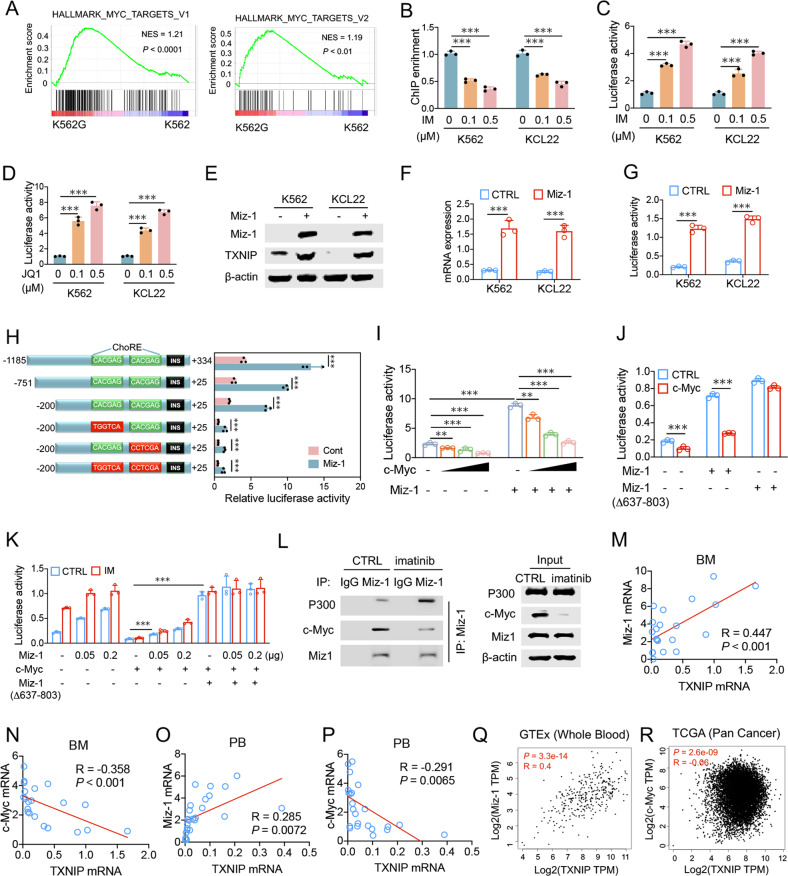
BCR-ABL blocks TXNIP transactivation by disrupting a Miz-1–p300 complex via c-Myc induction. **A** GSEA analysis of the RNAseq data predicts the gene enrichment in c-Myc targets in K562G and K562 cells. **B** The amount of c-Myc bound to the TXNIP promoter was determined by ChIP in K562 and KCL22 cells 24 h after imatinib treatment. **C**, **D** The activity of TXNIP promoter (−1185 ~ +334) was determined in K562 and KCL22 cells 24 h after imatinib (**C**) or JQ1 (**C**) treatment. **E**–**G** TXNIP protein (**E**), mRNA (**F**), and promoter activity (**G**) were determined, respectively, in K562 and KCL22 cells 48 h after the transfection of Miz-1 overexpression vector. **H** The indicated TXNIP promoter truncations were transfected into K562 cells with or without Miz-1 overexpression. The luciferase activity were determined. **I** K562 cells were transfected with the TXNIP reporter construct (−751 ~ +25) with or without a c-Myc expression plasmid. Increasing amounts (0.02, 0.1, and 0.2 μg) of Miz-1 construct were co-transfected as indicated. **J** K562 cells were transfected with the TXNIP reporter construct (−751 ~ +25) together with wild-type Miz-1 and its mutant construct, as indicated. **K** K562 cells were transfected with indicated constructs with TXNIP reporter truncation (−751 ~ +25). 24 h after transfection, cell were treated with 0.5 μM imatinib for additional 24 h. **L** K562 cells were transfected with Miz-1 constructs and treated with 0.5 μM imatinib. Immunoprecipitation was performed with Miz-1 antibody. Indicated proteins were further determined by western blot. **M**–**P** The mRNA expression associations of TXNIP with Miz-1 or c-Myc in the PB and BM were determined among samples from normal healthy donors and patients. Each individual dot represents 1 sample. **Q** The mRNA expression associations of TXNIP with Miz-1 were analyzed by GEPIA according to the whole blood samples from normal human in GETx dataset. **R** The mRNA expression associations of TXNIP with c-Myc were analyzed by GEPIA according to the Pan-Cancer samples from normal human in TCGA dataset. **B**, **C**, **K**, **L** IM, imatinib. ***P < 0.001. **P < 0.01.

TXNIP expression is subjected to the activation of key transcription factors, such as c-Myc [18] and MondoA [23]. Given that the inversed expression of c-Myc and TXNIP during either CML transformation or imatinib treatment (Fig. 1A, C, E), we suppose c-Myc is involved in BCR-ABL suppression of TXNIP transactivation in CML cells. In line with this, gene set enrichment analysis (GSEA) revealed that the enrichment of c-Myc target genes was dramatically increased in imatinib-resistant K562G cells (Fig. 2A). Whereas the enrichment of c-Myc on TXNIP promoter was decreased in CML cells after imatinib treatment (Fig. 2B). We previously demonstrated that c-Myc suppresses TXNIP transcription potentially through binding to the ChoRE element, which blocked MondoA-mediated TXNIP transactivation [18]. Whereas imatinib or c-Myc inhibitor (+)-JQ1 (JQ1) treatment can successively induce the activities of both wild type and ChoRE-mutant TXNIP promoter dose-dependently (Fig. 2C, D), indicating c-Myc mediated imatinib suppression of TXNIP is not predominantly through the competitive binding of c-Myc with MondoA on TXNIP promoter in CML cells.

Miz-1 is a transcription factor that transactivated a numerous gene expressions (e.g., p15^INK4b^ and p21^CIP1^) through the interaction with co-activator p300 [24, 25]. We here showed that the ectopic expression of Miz-1 in K562 and KCL22 cells induced the expressions of TXNIP protein and mRNA, and the promoter activity (Fig. 2E–G). Thus TXNIP potentially be a specific target of Miz-1 for transactivation. To further confirm this idea, we generated a series of truncations based on TXNIP promoter. These progressive deletions showed the core promoter of TXNIP was sufficient for activation by Miz-1 (Fig. 2H, I), and this effect was further enhanced with either imatinib treatment or overexpression of Miz-1△(637–803), which lacks the C-terminal residues (637–803) responsible for interaction with c-Myc (Fig. 2J, K). Co-expression of c-Myc reduced the basal activity of the TXNIP promoter and abrogated transactivation by Miz-1 or imatinib, but failed to block Miz-1△(637–803) activity (Fig. 2I, J). Co-immunoprecipitation (Co-IP) assay shows that imatinib treatment blocked the interaction of c-Myc with Miz-1, but enhanced Miz-1 and p300 interaction (Fig. 2L). Therefore, BCR-ABL blocks TXNIP transactivation by disrupting a Miz-1-p300 complex *via* c-Myc induction, which was compromised in CML cells with imatinib treatment. Supporting this idea, the expression of TXNIP was positively associated with Miz-1 and negatively associated with c-Myc in the samples from patients with myeloproliferative diseases (Fig. 2M–P). Accordingly, the whole blood samples from normal human in GETx dataset showed the positive association of Miz-1 with TXNIP (Fig. 2Q). The negative association of c-Myc with TXNIP in Pan-Cancer in TCGA dataset indicates that c-Myc generally suppresses TXNIP expression in different types of cancers (Fig. 2R).

### Suppression of TXNIP confers CML cell growth and counteracts imatinib sensitivity

**Fig. 3:**
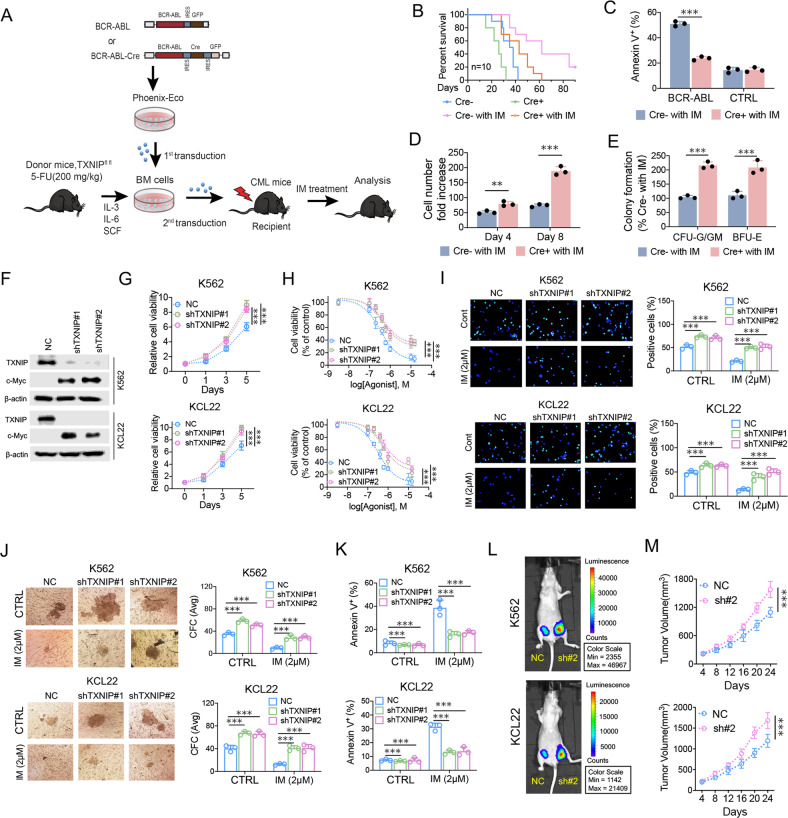
Suppression of TXNIP confers CML cell growth and counteracts imatinib sensitivity. **A** Schematic strategy of evaluation of the in vivo effect of TXNIP knockout on CML transformation. **B** Kaplan–Meier-style survival curves for recipients of BCR-ABL-Cre-GFP or BCR-ABL-GFP transduced BM cells from TXNIP^fl/fl^ (*n* = 10) mice. **C** Apoptosis of BCR-ABL or MIGR1 (CTRL) transduced normal CD34^+^ cells with or without Cre overexpression in the presence of 2 μM imatinib. The double-transduced CD34^+^ cells were sorted and then analyzed for annexin V + cells after 48 h of culture. **D**, **E** Analysis of total cell numbers (**D**) and colony-forming cell (CFC) formation (**E**) of BCR-ABL-Cre-GFP or BCR-ABL-GFP transduced normal CD34^+^ cells with 2 μM imatinib treatment. **F** The indicated protein levels were determined by western blot in K562 and KCL22 cells after TXNIP knockdown. **G** The cell viabilities of K562 and KCL22 cells were determined after TXNIP knockdown in indicated days. **H** The cell viability was determined in K562 and KCL22 cells following indicated doses of imatinib treatment for 72 h, and normalized to the control group. **I** Edu incorporation assay was performed for cell viability determination in K562 and KCL22 cells after TXNIP knockdown with or without 2 μM imatinib treatment. **J** K562 and KCL22 cells were plated in methylcellulose-containing medium after TXNIP knockdown, with or without imatinib treatment. The CFC were scored after 1 week. **K** Apoptosis was analyzed 48 h after imatinib treatment. **L** Representative bioluminescence imaging photographs of tumor burden in nude mice (*n* = 6) 24 days after the subcutaneous injection of indicated cells. sh#2: shTXNIP#2. **M** The Tumor size was measured and tumor volume was calculated by the formula (width^2^ × length × 0.5). IM: imatinib. ***P < 0.001. **P < 0.01.

To elucidate the function of TXNIP in malignant hematopoiesis, we firstly generated a mouse CML model with BM transduction and transplantation by using TXNIP conditional knock mice (TXNIP^fl/fl^) and BCR-ABL-Cre-GFP retrovirus (Supplementary Fig. 2A) [26]. We transduced bone marrow cells from TXNIP^fl/fl^ mice with BCR-ABL-Cre-GFP or BCR-ABL-GFP retrovirus, followed by transplantation of the transduced cells into lethal irradiated recipient mice (Fig. 3A). Mice receiving donor bone marrow (BM) cells transduced with BCR-ABL-Cre-GFP developed CML much faster than those receiving bone marrow cells transduced with BCR-ABL-GFP, and further blocked imatinib effect on survival of CML mice (Fig. 3B). Thus, TXNIP is a potent tumor suppressor in BCR-ABL-induced CML. Supporting this idea, TXNIP knockout BM cells showed decreased apoptosis after imatinib treatment, but had a minimal effect on normal CD34^+^ cells (Fig. 3C). In accordance, TXNIP knockout increased BM cell proliferation and colony-forming cell (CFC) formation (Fig. 3D, E).

Furthermore, we determined TXNIP tumor suppressive function in the CML cell lines by lentivirus expressing TXNIP shRNA (Fig. 3F). TXNIP knockdown dramatically increased CML cell growth rates, which were confirmed by either CCK8 and Edu incorporation assays (Fig. 3G–I). Particularly, TXNIP reduction counteracted the inhibitory effect of imatinib on cell survival in both K562 and KCL22 cells (Fig. 3H, I). In accordance, TXNIP knockdown resulted in significantly larger colonies and the induction in the CFC -formation relative to the control CML cells (Fig. 3J). Thus, suppression of TXNIP contributes to the increased cell activity and decreased imatinib effect on CML cells. Supporting this idea, imatinib treatment lead to the death of CML cells, and the increased cell death were compromised by TXNIP knockdown (Fig. 3K). Further, the mice with TXNIP-decreased CML cells developed tumors more quickly than the control group (Fig. 3L, M and Supplementary Fig. 3A). In the related tumor section, the proliferation marker Ki-67 is increased after TXNIP suppression (Supplementary Fig. 3B, C).

Thus, TXNIP is necessary for imatinib suppressive effect on CML cell growth, and the blockage of TXNIP expression results in the increased CML cell survival and compromises imatinib effect.

### TXNIP compromises imatinib-resistant CML cell growth and CML transformation

**Fig. 4:**
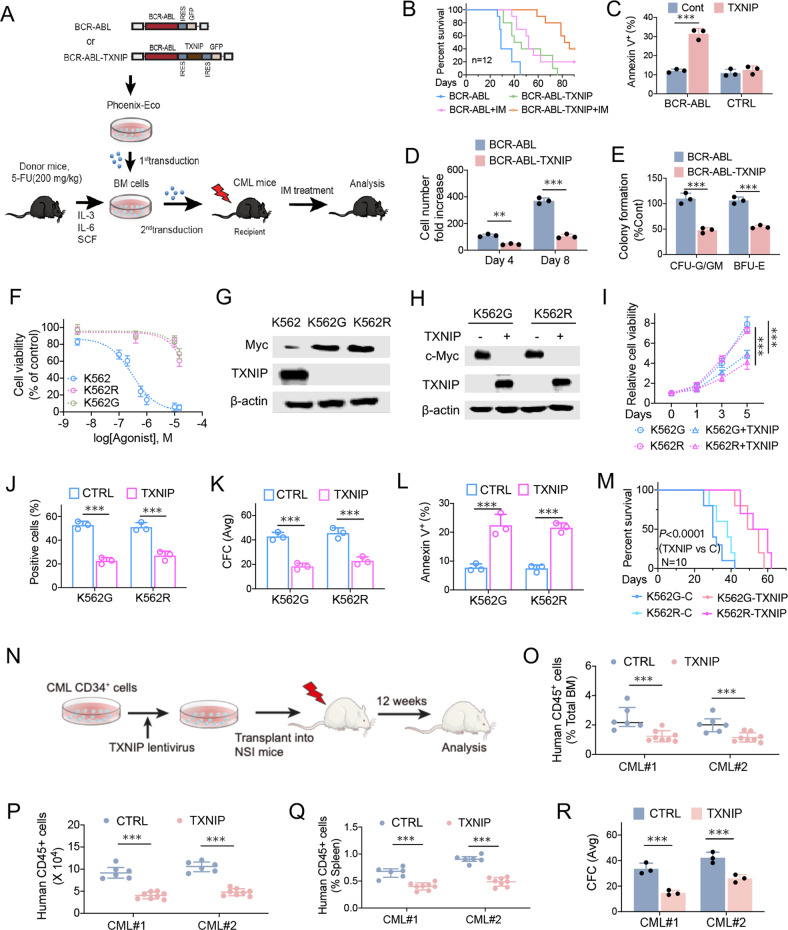
TXNIP compromises imatinib-resistant CML cell growth and CML transformation. **A** Schematic strategy of evaluation of the in vivo effect of TXNIP overexpression on CML transformation. **B** Kaplan–Meier-style survival curves for recipients of BCR-ABL-TXNIP-GFP or BCR-ABL-GFP transduced BM cells from C57BL/6 (*n* = 12) mice. **C** Apoptosis of BCR-ABL or MIGR1 (CTRL) transduced normal CD34^+^ cells with or without TXNIP overexpression. The double-transduced CD34^+^ cells were sorted and then analyzed for annexin V + cells after 48 h of culture. **D**, **E** Analysis of total cell numbers (**D**) and CFC formation (**E**) of BCR-ABL-TXNIP-GFP or BCR-ABL-GFP transduced normal CD34^+^ cells. **F** The cell viability was determined in K562, K562G, and K562R cells following indicated doses of imatinib treatment for 72 h, and normalized to the control group. **G** TXNIP and c-Myc expressions in K562, K562G, and K562R cells. **H** TXNIP and c-Myc expressions in TXNIP overexpressed K562G and K562R cells. **I**–**L** In TXNIP overexpressed K562G and K562R cells, the cell viabilities were determined by CCK8 (**I**) and Edu staining (**J**) assay. The CFC formation (**K**) and apoptosis (**L**) were analyzed, respectively. **M** Survival curves of mice receiving xenografted CML cells. K562G or K562R cells with or without TXNIP overexpression were inoculated into nude mice. Mice were euthanized when the tumor volume reached 1000 mm^3^. **N** The human primary CML CD34^+^ cells were infected with TXNIP lentivirus, and then injected into sublethally irradiated (300 cGy) NSI mice. After 12 weeks, human multilineage engraftment was analyzed by flow cytometry. **O**, **P** The percentage (O) and the absolute number (**P**) of human CD45^+^ cells engrafted in the BM after transplantation of human CML CD34^+^ cells (1 × 10^6^ cells/mouse) for 12 weeks. **Q** The proportion of human CD45^+^ cells engrafted in the spleen at 12 weeks. **R** The CFC formation after TXNIP overexpression. ***P < 0.001. **P < 0.01.

We further generated BCR-ABL-TXNIP-GFP co-expressing retrovirus to determine the highly expression of TXNIP in CML development (Supplementary Fig. 2B). C57BL/6 donor BM cells were transduced with BCR-ABL-TXNIP-GFP or BCR-ABL-GFP retrovirus, followed by transplantation of the transduced cells into recipient mice (Fig. 4A). CML development was significantly slower in mice receiving BM cells transduced with BCR-ABL- TXNIP-GFP than in those receiving BM cells transduced with BCR-ABL-GFP (Fig. 4B). Further, TXNIP expression in BM cells increased apoptosis, and suppressed proliferation and CFC formation (Fig. 4C–E). These data indicate that TXNIP overexpression caused a delay of CML development.

We further generated imatinib-resistant K562R cells, which is through the short-term induction of K562 cells with low dose of imatinib treatment, to determine the association of drug resistance of CML with TXNIP expression (Fig. 4F). Compared with parental K562 cells, TXNIP was dramatically decreased in both K562G and K562R cells (Fig. 4G), indicating altered expression of TXNIP is an early event during the resistance of CML to imatinib. We therefore restored TXNIP expression by lentivirus in both K562G and K562R cells (Fig. 4H). The ectopic expression of TXNIP suppressed CML cell growth and CFC formation, and resulted in the increased apoptosis in K562G and K562R cells (Figure 4I–L). The growth of xenografted CML cells were also diminished in accordance (Fig. 4M). We also determined TXNIP overexpression in human CML CD34^+^ cells on their ability to be engrafted in NOD-*scid Il2rg*−*/−* (NSI) mice (Fig. 4N). TXNIP expression reduced the engraftment of human CML CD45^+^ cells in BM (Fig. 4O, P) and spleen (Fig. 4Q) at 12 weeks after transplantation. Consistently, TXNIP expression in human CML cells suppressed cell colony formation (Fig. 4R). Therefore, TXNIP compromises CML transformation and development. The induction of TXNIP expression could be considered as a strategy for the treatment of CML patients with imatinib-resistance.

### BCR-ABL triggers a glucose-dependent survival program *via* TXNIP suppression

**Fig. 5:**
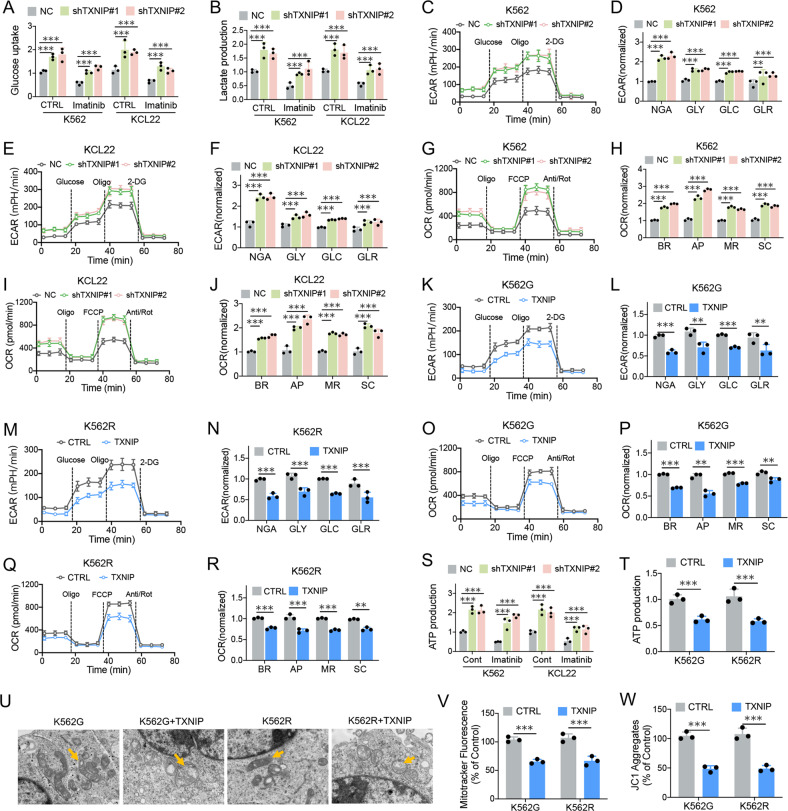
BCR-ABL triggers a glucose-dependent survival program via TXNIP suppression. **A** 2 NBDG assays for glucose uptake in K562 and KCL22 cells with TXNIP knockdown 24 h after 1 μM imatinib treatment. **B** Lactate production in K562 and KCL22 cells with TXNIP knockdown 24 h after 1 μM imatinib treatment. **C**–**F** The high throughput Seahorse assays to monitor extracellular acidification rate (ECAR) in K562 (**C**, **D**) and KCL22 (**E**, **F**) cells with TXNIP knockdown. **G**–**J** The high throughput Seahorse assays to monitor cellular oxygen consumption rate (OCR) in K562 (**G**, **H**) and KCL22 (**I**, **J**) cells with TXNIP knockdown. **K**–**N** The high throughput Seahorse assays to monitor ECAR in K562G (**K**, **L**) and K562R (**M**, **N**) cells with TXNIP overexpression. **O**–**R** The high throughput Seahorse assays to monitor cellular OCR in K562G (**O**, **P**) and K562R (**Q**, **R**) cells with TXNIP overexpression. **S**, **T** The intracellular ATP concentrations were determined in indicated cells with TXNIP knockdown after 1 μM imatinib treatment (**S**), or TXNIP overexpression (**T**). **U** The transmission electron microscope assays for assessing the mitochondrial shape in K562G and K562R cells after TXNIP overexpression. **V** MitoTracker-based flow cytometry assays for assessing mitochondrial number. **W** JC1-mitochondrial membrane potential assays. BR basic respiration, AP ATP production, MR maximal respiration, SC spare capacity, NGA non-glycolytic acidification, GLY glycolysis, GLC glycolytic capacity, GLR glycolytic reserve. ***P < 0.001. **P < 0.01.

Numerous studies demonstrated that BCR-ABL activates glucose metabolism as part of its transforming activity in CML [10]. The glycolytic metabolism is suggested to play a key role in determining imatinib efficacy and provides a rationale for targeting glycolytic metabolism therapeutically [12]. In accordance, our study shows that imatinib treatment compromised the rates of glucose uptake and lactate production in both K562 and KCL22 cells (Fig. 5A). Due to the key role of TXNIP in regulating glucose metabolism for the suppression of cell survival, we determined TXNIP effect on BCR-ABL triggered glucose metabolic reprogramming in CML cells. Strikingly, TXNIP knockdown in CML cells increased glucose uptake and lactate production which were suppressed by imatinib (Fig. 5A, B), indicating its inhibitory role in glycolysis. Further, TXNIP knockdown counteracted imatinib inhibitory effect on glucose metabolism, including glycolysis (including non-glycolytic acidification, glycolysis, glycolytic capacity, and glycolytic reserve) and mitochondrial respiration (including lower levels of basal respiration, ATP production, maximal respiration, and spare capacity), in both K562 and KCL22 cells (Fig. 5C–J). In imatinib-resistant K562R and K562G cells, TXNIP overexpression diminished glucose uptake and lactate production, and further blocked glycolysis and mitochondrial respiration process (Fig. 5K–R). Accordingly, the internal ATP levels were restored after TXNIP knockdown in imatinib-treated CML cells, and decreased after TXNIP overexpression (Fig. 5S, T). Moreover, the transmission electron microscope revealed that TXNIP overexpression resulted in mitochondrial swelling and loss and lysis of mitochondrial crest in K562G and K562R cells (Fig. 5U). MitoTracker and JC1-based flow cytometry assays further demonstrated that the presence of TXNIP decreased mitochondrial mass and mitochondrial membrane potential (Fig. 5V, W), suggesting TXNIP suppression of glucose metabolism confers mitochondrial dysfunction in imatinib-resistant CML cells. Therefore, BCR-ABL-mediated TXNIP suppression results in the increased glucose utilization, and the resumed TXNIP expression increased imatinib efficacy predominantly through the blockage of glucose metabolism and mitochondrial function.

### TXNIP suppresses HK2 and LDHA expressions through Fbw7-dependent c-Myc degradation

**Fig. 6:**
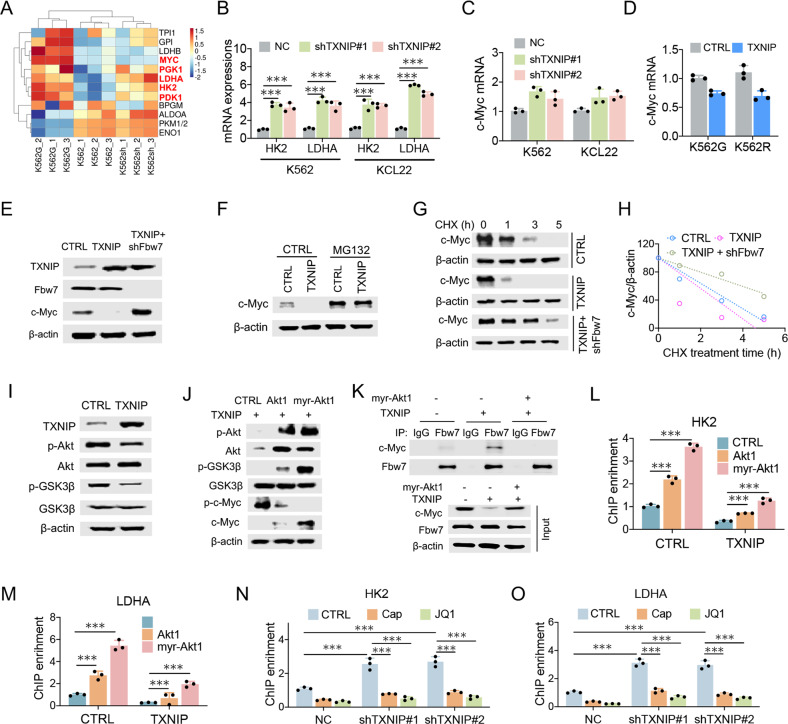
TXNIP suppresses HK2 and LDHA expressions through Fbw7-dependent c-Myc degradation. **A** The heatmap represents the expression of glycolytic-related genes in K562 cells, K562 cells with TXNIP knockdown (K562sh), and K562G cells according to the RNAseq data. **B** HK2 and LDHA mRNA levels in K562 and KCL22 cells with or without TXNIP knockdown. **C**, **D** c-Myc mRNA levels in indicated cells with TXNIP knockdown or overexpression. **E** Indicated protein levels were determined in K562 cells with or without TXNIP overexpression, or TXNIP-overexpressed K562 cells with Fbw7 knockdown. **F** K562 cells with or without TXNIP expression were treated with 20 μM MG132 for 2 h. Levels of indicated proteins were determined by western blot. **G** Levels of indicated proteins in indicated K562 cells following 10 μg/ml CHX treatment for indicated time. **H** c-Myc expression levels were further quantified by ImageJ and normalized to β-actin to determine the protein degradation rate. **I** Levels of indicated proteins in K562 cells with or without TXNIP overexpression. **J**–**M** We overexpressed the wild type or activating form of Akt1 (myr-Akt1) in TXNIP overexpressed K562 cells. **J** The indicated proteins levels were determined by western blotting. **K** Immunoprecipitation was performed to determine the interaction between c-Myc and Fbw7 in indicated K562 cells. After the Fbw7 protein were immunoprecipitated with an anti-Fbw7 antibody, indicated proteins were detected by western blotting. **L**, **M** c-Myc enrichment on HK2 (**L**) and LDHA (**M**) promoter were determined by ChIP. **N**, **O** c-Myc enrichment on HK2 (**N**) and LDHA (**O**) promoter were determined by ChIP in K562 cells with or without TXNIP knockdown after AKT inhibitor Capivasertib (Cap) or c-Myc inhibitor JQ1 treatment. ***P < 0.001.

To fully address the underlying mechanism of TXNIP tumor suppressive effect on regulating glucose metabolism, RNA sequencing was performed in K562 cells after TXNIP knockdown. Intriguingly, the key glycolytic enzymes, HK2 and LDHA, were increased in TXNIP-suppressed K562 cells, in line with their elevated expressions in imatinib-resistant K562 cells (Fig. 6A, B). Combined with the data of elevated c-Myc expression in K562 cells with TXNIP knockdown and decreased c-Myc in TXNIP overexpressed K562 cells (Figs. 3F and 4H), TXNIP suppression of glycolytic enzyme expressions potentially depends on compromising c-Myc transcriptional activity. Moreover, the dramatically alterations of c-Myc protein levels by TXNIP potentially not be attribute to the minimal variation of c-Myc mRNA abundance (Fig. 6C, D), raising the possibility that TXNIP regulates c-Myc in post-translational level. Supporting this idea, c-Myc stability was attenuated in TXNIP-overexpressed K562G cells (Fig. 6E–H). Fbw7 (F-box and WD repeat domain-containing 7) is an E3 ubiquitin ligase and targets many substrates for proteasomal degradation, including c-Myc [27]. Knockdown Fbw7 restored c-Myc protein expression and stability which were repressed by TXNIP (Fig. 6E–H). Thus, TXNIP represses c-Myc potentially through the induction of Fbw7-dependent c-Myc degradation, which results in the suppression of glycolysis in CML cells.

Numerous studies showed that GSK3β facilitates c-Myc turnover through promoting c-Myc (Thr58) phosphorylation, and subsequently promotes c-Myc for ubiquitination and degradation by Fbw7 [27, 28]. Whereas Akt activation stabilizes c-Myc through phosphorylating GSK3β Ser9, which is associated with GSK3β inactivation. We assume that TXNIP suppression of c-Myc stabilization is mediated by AKT/GSK3β pathway. Notably, TXNIP expression decreased Akt activity (Fig. 6I). Thus we recovered Akt activity by the ectopic expression of either Akt1 or a constitutively active Akt1 that contained a myristoylation sequence (myr-Akt1). The remarkable increase of c-Myc expression and decrease of c-Myc (T58) phosphorylation were observed after Akt1 or myr-Akt1 overexpression in TXNIP-overexpressed K562G cells (Fig. 6J). Particularly, TXNIP expression increased the interaction of c-Myc with Fbw7, which was blocked by Akt activation (Fig. 6K). c-Myc directly activates HK2 and LDHA transcription through the direct binding on promoter regions of HK2 and LDHA contain E-box (5’-CACGTG-3’) [29]. Chromatin immunoprecipitation (ChIP) with c-Myc antibody demonstrates that the enrichment of c-Myc on HK2 and LDHA promoter was decreased by TXNIP, and restored after Akt activation (Fig. 6L, M). Further, the increased of c-Myc enrichment by TXNIP knockdown can be blocked by either Akt inhibitor Capivasertib or c-Myc inhibitor JQ1 (Fig. 6N, O). Therefore, TXNIP suppresses c-Myc expression via the inhibition of Akt activation which is associated with Fbw7-depdent c-Myc degradation, and consequently blocks c-Myc-dependent glycolytic enzyme expressions.

### Combination of TXNIP induction with imatinib for treatment of CML

**Fig. 7:**
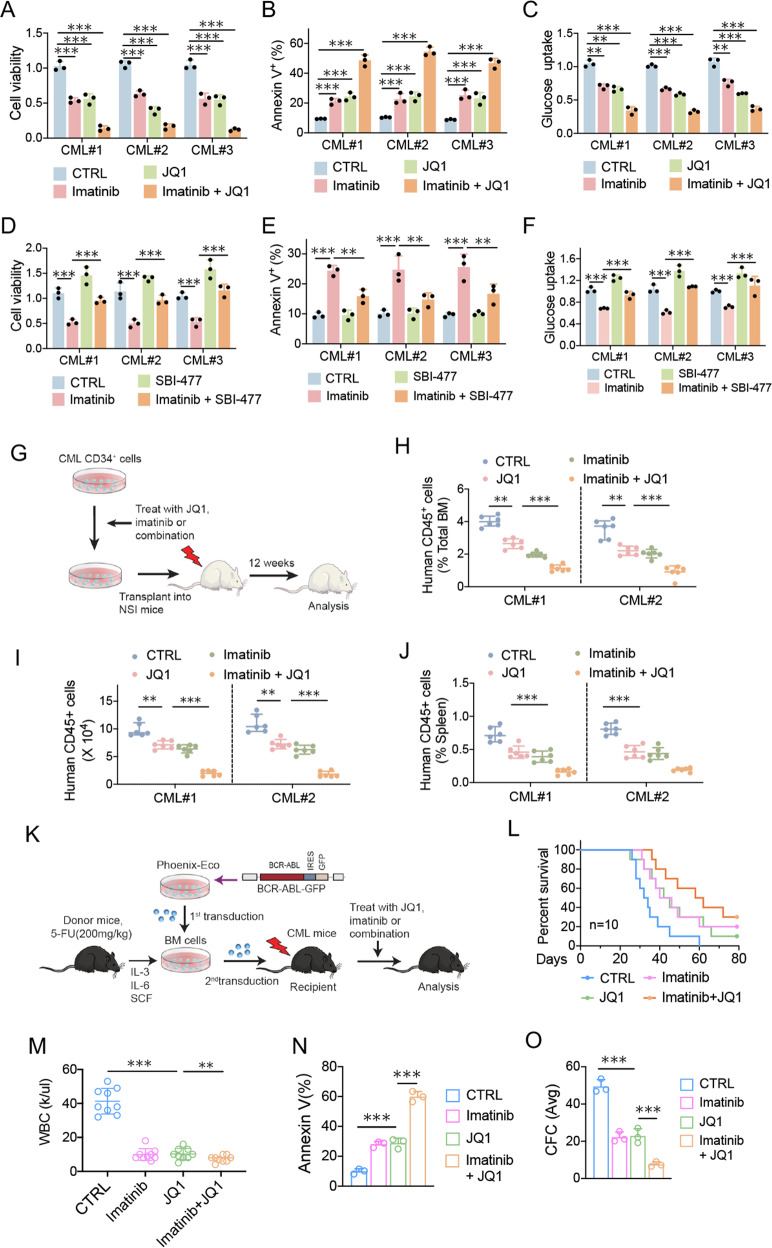
Combination of TXNIP induction with imatinib for treatment of CML. **A**–**C** The primary CML cells (#1, #2, #3) from patients were cultured and treated with 0.5 μM JQ1, 1 μM imatinib, or combination. **A** Cell viabilities were determined by CCK8 assay 72 h after drug treatment. **B** Apoptosis was analyzed 48 h after drug treatment. **C** 2-NBDG for glucose uptake was measured 24 h after drug treatment. **D**–**F** The primary CML cells (#1, #2, #3) from patients were cultured and treated with 3 μM SBI-477, 1 μM imatinib, or combination. **D** Cell viabilities were determined by CCK8 assay 72 h after drug treatment. **E** Apoptosis was analyzed 48 h after drug treatment. **F** 2-NBDG for glucose uptake was measured 24 h after drug treatment. **G** The human primary CML CD34^+^ cells were treated with JQ1, imatinib or the combination for 72 h, and then injected into sublethally irradiated (300 cGy) NSI mice. After 12 weeks, human multilineage engraftment was analyzed by flow cytometry. **H**, **I** The percentage (**H**) and the absolute number (**I**) of human CD45^+^ cells engrafted in the BM after transplantation of human CML CD34^+^ cells (1 × 10^6^ cells/mouse) for 12 weeks. **J** The proportion of human CD45^+^ cells engrafted in the spleen at 12 weeks. **K** Schematic strategy of evaluation of the in vivo effect of indicated drugs on CML transformation. **L**, **M** Survival curves (**L**) and total blood leukocyte counts (**M**) for CML mice treated with indicated drugs. Ten days after receiving 1 × 10^5^ BM cells transduced by BCR-ABL-GFP, mice were treated by imatinib (200 mg/kg/d), JQ1 (50 mg/kg/d), or combination for 10 days. Total blood leukocyte counts were analyzed at day 20 after transplantation. **N** BM cells from CML mice were cultured with 2 μM imatinib, 0.5 μM JQ1, or combination for 48 h, and apoptosis was analyzed for GFP^+^ cell fraction. **O** 24 h after drug treatment, BM cells were plated in methylcellulose medium for CFC assay. ***P < 0.001. **P < 0.01.

To determine whether TXNIP synergizes with imatinib in producing growth inhibition for CML treatment, we introduced the drug JQ1 (TXNIP activator) and SBI477 (TXNIP inhibitor). In the primary CML cells, JQ1 treatment suppressed cell survival and induced apoptosis, and these effects were augmented in combination with imatinib (Fig. 7A, B). The decreased glucose uptake rates by JQ1 and imatinib indicate that synergistic inhibitory effect is predominantly due to the suppression of glucose metabolism (Fig. 7C). In accordance, TXNIP inhibitor SBI477 counteracted imatinib mediated suppression of cell survival and glucose uptake and induction of apoptosis in primary CML cells (Fig. 7D–F). Furthermore, we determined the effect of ex vivo treatment with JQ-1, imatinib or combination in human primary CML cells on their ability to be engrafted in NSI mice (Fig. 7G). JQ1 treatment reduced the engraftment of human CML CD45^+^ cells(Fig. 7H, I) and spleen (Fig. 7J) at 12 weeks after transplantation, and sensitized imatinib effect.

To further confirm this combination, we generated a mouse CML-like disease model (Fig. 7K). JQ1 significantly reduced white blood cells (WBC) counts and extended the survival of CML mice (Fig. 7L, M). No obvious hematologic toxicity or body weight loss was detected with tenovin-6 alone or in combination with imatinib (Supplementary Fig. 4). Imatinib extended the survival of CML mice in a manner similar to JQ1, whereas JQ1 plus imatinib treatment provided a further survival advantage for CML mice (Fig. 7L). Further, BM cells from CML mice were isolated and analyzed for their sensitivity to both drugs in vitro. JQ1 sensitized mouse cells to imatinib-induced apoptosis and further suppressed CFC formation (Fig. 7N, O). These results indicate that JQ1 enhances imatinib treatment potentially through the induction of TXNIP which results in the blockage of glucose metabolism during CML disease development.

## Discussion

**Fig. 8:**
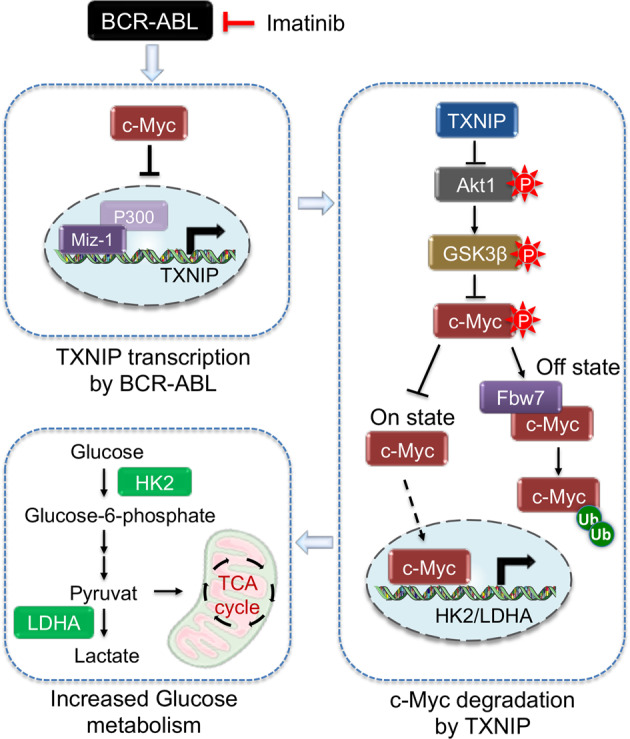
Schematic drawing of the mechanism by which BCR-ABL signaling triggered glucose-dependent survival program through TXNIP suppression by regulating c-Myc/Miz1 complex. TXNIP suppresses HK2 and LDHA expressions through accelerating Fbw7-dependent c-Myc degradation by the phosphorylation of Akt/GSK3β axis.

Growing evidence has demonstrated that malignant transformation of myeloid and lymphoid cells by BCR-ABL involves the dysregulation or mutation of a variety of genes that are normally involved in regulating the proliferation and survival of hematopoietic cells [30–32]. The identification of BCR-ABL suppression of TXNIP increases our understanding of CML and expands the previously known BCR-ABL-regulated molecular network [2]. TXNIP restoration sensitizes CML cells to imatinib treatment, potentially through the blockage of glucose metabolism. Mechanistically, TXNIP expression was decreased in response to the activated BCR-ABL signaling, which is associated with a previously unappreciated mechanism that involves in c-Myc/Miz-1/P300 complex. Whereas, TXNIP suppresses glycolytic enzyme expressions through Fbw7-dependent c-Myc degradation, indicating a positive feedback loop between c-Myc and TXNIP. BCR-ABL suppression of TXNIP provides a novel survival pathway for CML transformation (Fig. 8). In particular, combination of TXNIP induction with imatinib effectively suppresses the development of CML-like myeloproliferative disease in mice, which provides a rationale for treating patients with CML with minimal residual disease.

BCR-ABL signaling engages a glucose-dependent survival program in CML cells. Increased glucose transporter expression, glucose uptake and lactate production have been previously reported in BCR-ABL + cells, and resistance to tyrosine kinase inhibitors is correlated with sustained glucose metabolism [12, 13]. Particularly, inhibition of glycolysis can enhance the imatinib cytotoxicity [10]. Thus glycolytic metabolism is suggested to play a key role in determining imatinib efficacy and provides a rationale for targeting glycolytic metabolism therapeutically. Our work identified that BCR-ABL-mediated glucose utilization is dependent on TXNIP, which has previously been confirmed as a potent negative regulator of glucose uptake and aerobic glycolysis by our and other groups [18–20]. TXNIP expression decreased in chronic CML progenitor cells and barely detectable in the advanced phases of CML. Suppression of TXNIP in CML cells restored glucose uptake and glycolytic rate with was suppressed by imatinib, and subsequently provided a survival advantage and drug resistance of CML cells.

c-Myc, subjected to BCR-ABL signaling, is a multifunctional transcription factor necessary for rapid cell division and simultaneously inhibits the expression of genes with antiproliferative functions [33, 34]. Due to c-Myc suppresses TXNIP transcription through the competition of ChoRE region with MondoA, we suppose BCR-ABL suppresses TXNIP via c-Myc activation and further confirmed this idea. However, the successful induction of TXNIP promoter activity by imatinib indicates that other factors is involved in BCR-ABL-triggered TXNIP suppression. Further studies demonstrated that Miz-1 can specifically induce TXNIP transactivation in CML cells, in response to BCR-ABL inhibition by imatinib. The core promoter of TXNIP can be activated by Miz-1, and this effect was further enhanced with overexpression of Miz-1△(637–803), which lacks the motif responsible for c-Myc interaction. In addition, imatinib treatment blocked the interaction of c-Myc with Miz-1, but enhanced Miz-1 and p300 interaction. Thus we point out that a new route of c-Myc-mediated TXNIP suppression in CML cells, that BCR-ABL blocks TXNIP transactivation by disrupting a Miz-1–p300 complex via c-Myc induction, which was compromised in CML cells with imatinib treatment. The clinical relevance further supported this idea. Further studies are needed to confirm the relevance of this regulation to other tissues.

Drug resistance and malignant transformation involve cellular metabolic changes, which may in turn render the cells susceptible to specific assaults in a selective manner [35]. The elevated glucose uptake and glycolytic rate of imatinib-resistant CML cells has already been identified in previous reports [12, 13, 36]. The long-term treatment of imatinib results in numerous gene mutations, such as T315I mutation in BCR‑ABL, which confer CML cell resistance in response to imatinib [37, 38]. However, this cannot fully address how resistant cells favor glycolysis. In this study, we generated imatinib-resistant K562 cells through either long-term (K562G) or short-term (K562R) imatinib induction. We observed that TXNIP expression is dramatically reduced in imatinib-resistant K562 cells, indicating the increased glucose metabolism potentially be attribute to the reduced TXNIP expression. Particularly, TXNIP reduction occurs in both K562G and K562R cells, indicating TXNIP-mediated glucose metabolic reprogramming is an early event during the resistance of CML to imatinib. The increased glycolysis prefers as a trigger of imatinib resistance to as a effector, in response to the decreased TXNIP expression in CML cells.

The tumor suppressive role of TXNIP through negative regulating glucose metabolism has been reported broadly [39–41]. Although TXNIP-mediated GLUT1 trafficking and internalization was identified [22], whether and how TXNIP regulates a large scale of glycolytic enzyme expressions regrading to the inhibition of glucose metabolism is largely unknown. We here demonstrate that TXNIP suppresses glycolytic enzyme expressions potentially through Fbw7-dependent c-Myc degradation, which provides a novel mechanism of TXNIP-dependent glucose inhibition. It is widely accepted that elevated c-Myc expression is dependent on the aberrant activation of PI3K/Akt signaling. Akt inactivates GSK3β through the phosphorylation at Ser 9 of GSK3β, which results in the suppression of GSK3β-mediated c-Myc (T58) phosphorylation and Fbw7-dependent c-Myc degradation [27, 28]. Supporting this idea, we identified that TXNIP suppressed the phosphorylated Akt and GSK3β levels, and increased c-Myc phosphorylation and Fbw7-dependent degradation in advance. Further, the increased c-Myc enrichment on HK2 or LDHA promoter by TXNIP knockdown can be blocked by either Akt inhibitor Capivasertib or c-Myc inhibitor JQ1. Thus TXNIP suppresses c-Myc expression via the inhibition of Akt activation which is associated with Fbw7-depdent c-Myc degradation, and consequently blocks c-Myc-dependent glycolytic enzyme expressions, suggesting a positive feedback loop between c-Myc and TXNIP. In line with this finding, subjective evidence have demonstrated TXNIP-dependent inactivation of PI3K/Akt pathway [42, 43].

Since the key role of TXNIP in increasing imatinib sensitivity and counteracting drug resistance in CML cells, it is reasonable to determine the synergistic inhibitory effect of imatinib with drugs targeting TXNIP. JQ1 is an inhibitor of Brd4, which belongs to the bromodomain and extra-terminal domain (BET) family. We and others previously identified that JQ1 transactivates TXNIP [18, 44], and TXNIP is supposed to be a pharmacodynamics biomarker of for targeting Brd4 [45]. We demonstrate the effective cell growth suppression during combination of JQ1 with imatinib for treatment of either the primitive CML samples and the mouse CML-like disease model. In line with the previous report that JQ1 cooperates with BCR/ABL TKI in inducing Ph^+^ CML growth-inhibition [46], we further point out that the synergistic inhibitory effect is predominantly due to the suppression of TXNIP. Supporting this idea, TXNIP suppression with SBI477 counteracted imatinib-mediated suppression of cell survival in the primitive CML samples.

Taken together, our study provides a new route of BCR-ABL signaling triggered glucose-dependent survival program, which is predominantly through the suppression of TXNIP by regulating c-Myc/Miz1 complex. TXNIP augments imatinib effects and overcome resistance through suppressing glycolysis in a Fbw7-dependent c-Myc degradation manner. The induction of TXNIP expression by JQ1 synergizes with imatinib in producing growth inhibition in CML cells. Thus targeting TXNIP is a potent approach to treating patients with CML and overcoming drug resistance.

## Materials and methods

### Cell lines and DNA constructs

K562 and KCL-22 cells were purchased from Cell Bank of the Type Culture Collection of the Chinese Academy of Sciences, Shanghai, China. Imatinib-resistant K562/G01 (K562G) and K562R cells were generated as previously described [47]. TXNIP overexpression and knockdown lentivirus was packaged in Hanheng Biotechnology, Shanghai, China. The TXNIP-luciferase reporter constructs and shRNA sequence were described previously [18, 21]. Luciferase overexpression lentivirus were packaged in Hanheng Biotechnology, Shanghai, China. Fbw7 siRNA was purchased from ThermoFisher (#133652).

### Human samples and isolation

The study of human samples was approved by the ethics committee of the Fourth Military Medical University (No. 202003-146). Samples of bone marrow (BM) and peripheral blood (PB) cells collected from normal healthy donors and patients with AML or CML were provided from Tangdu hospital, the fourth military medical university of China. For this study, Mononuclear cells were isolated by Ficoll-Paque (GE Healthcare, 17,144,003) density-gradient centrifugation. CD34^+^ cells were isolated and cultured using a positive magnetic bead selection protocol (StemCell Technologies) as previously described [48].

### RNA seq analysis and bioinformatics

The RNA were extracted from K562 cells with indicated treatment. Paired-end libraries were synthesized by using the TruSeq™RNA sample preparation kit (Illumina, USA). Following purification, the mRNA was fragmented into small pieces and the cleaved RNA fragments were conversed to cDNA. The products were then purified and enriched with PCR to create the final cDNA library. After cluster generation, the libraries were sequenced using an Illumina NovaSeq 6000 platform (Illumina, USA). Fragments per kilobase of exon per million reads mapped (FPKM) was quantified using StringTie and ballgown. Significant differentially expressed RNAs with the absolute value of fold change 1.5 and *p* value <0.05 were annotated to GO terms and KEGG pathways. The GSEA analysis (http://www.broad.mit.edu/gsea/index.html) was performed based on the known target genes in the MsigDB gene sets.

### Quantitative PCR

The RNA was extracted and cDNA was generated through GoScript Reverse Transcription System (Promega). The quantitative PCR (qPCR) assay was performed as described previously [49]. Primer sequences are listed in Supplementary Table 1.

### Chromatin immunoprecipitation

The chromatin immunoprecipitation (ChIP) analysis was performed using the ChIP Assay kit (Upstate Biotechnology, Charlottesville, VA, USA) as described previously [50]. Briefly, One 15-cm plate of CML cells was used for each condition. Crosslinked and sheared chromatin was prepared incubated overnight at 4 °C with 2 μg anti-Miz1 or anti-c-Myc antibodies. Immunocomplexes were captured with 10 μL Dynabeads M-280 sheep anti-rabbit/mouse (Invitrogen) for 1 h at 4 °C. After washing, reversal of crosslinks, and DNA purification, transcription factor binding was determined by normalizing to input or input + IgG controls and was expressed relative to the highest enriched condition. The sequences of PCR primers are listed in Supplementary Table 2.

### Immunoprecipitation and Western blotting

Cells were harvest and extracted. The extracts were incubated with anti-Fbw7 antibody or anti-Miz-1 antibody overnight at 4 °C. After blended with Protein A/G Dynabeads® (Thermofisher), the complex was washed twice with PBS, in turn to be resuspended and boiled with 2 × SDS loading buffer and subjected to Western blot analysis. Western blotting assay was performed as previously described [49]. The detailed information of antibodies were listed in Supplementary Table 3.

### Luciferase reporter assay

TXNIP promoter truncations of different lengths or mutations were constructed with pGL4 promoter. Luciferase assays were performed using the dual luciferase reporter assay system (Promega). The values are expressed as the means ± s.d. of at least three independent experiments.

### Cell viability and apoptosis assays

For Cell Counting Kit-8 (CCK-8) assay, cells were seeded at a density of 1000 cells/well in 96-well plates with indicated treatment. Then, 10 μl of CCK-8 solution (Targetmol C0005, China) were added to each well and incubated with the cells for 4 h at 37 °C. The absorbance at 450 nm was measured by a microplate spectrophotometer BioRad, Philadelphia, PA, USA). For EdU proliferation assay, cells were seeded in 24-well plates with complete media. Cell proliferation was detected using the incorporation of 5-ethynyl-20-deoxyuridine (EdU) with the EdU Cell Proliferation Assay Kit (Ribobio, Guangzhou, China) according to the manufacturer’s protocol. The cell nuclei were stained with DAPI (Sigma) at a concentration of 1 mg/ml for 20 min. The proportion of the cells incorporated EdU was determined with fluorescence microscopy. For in vitro Colony Forming Unit Assays: Cells were collected, and 1 × 10^3^ cells were transferred to Methocult medium (MethoCult GF H4435, StemCell Technologies). The number of colonies was counted 1 week after plating. For assays of apoptosis, the cells were incubated for 15 min with annexin V (BD Biosciences) and then analyzed by flow cytometry.

### Glucose uptake assay

Cells were grown in 6-well plates until reaching 80–90% confluence and glucose uptake was determined by adding 2-(N-(7-nitrobenz-2-oxa-1,3-diazol-4-yl)amino)-2-deoxyglucose (2-NBDG, Sigma) to the cell culture medium at 50 μM for 30 min. Then cells were centrifuged at 800 rpm for 5 min and resuspended in PBS supplemented with 2% FBS for flow cytometric analysis. Fluorescence was measured using the 488 nm excitation and 530 nm emission wavelengths to detect FITC under the flow cytometry.

### Lactate and ATP production assay

Cells were (1.0 × 10^5^) were incubated overnight on 6-well plates, intracellular lactate and ATP production were determined with Lactate Colorimetric/Fluorometric Assay Kit (Biovision, Abcam) and ATP Bioluminescence Assay Kit CLS II (Roche Applied Science) according to the manufacturer’s protocol, respectively. The relative OD values were measured using the microplate reader.

### Mitochondrial related assays

To assess the mitochondrial membrane potential and mitochondrial mass, K562G and K562R cells with or without TXNIP knockdown were stained with the JC-1 reagent (2 μM, #HY-15534, MedChemExpress) and MitoTracker Green (0.1 μM, #HY-135056, MedChemExpress) at 37 °C for 30 min, respectively. Then, cells were subjected to flow cytometric analysis. To visualize the mitochondrial shape, cells were fixed within 3% glutaraldehyde, postfixed with 1% osmium tetroxide followed by 2% uranyl acetate, dehydrated through a series of ethanol gradients, and embedded. Ultrathin sections were isolated on nickel fitters, stained with uranyl acetate followed by lead citrate, and viewed under the transmission electron microscope.

### Protein degradation assay

TXNIP-overexpressing K562 cells were incubated with CHX (10 μg/ml, Sigma) for indicated time. Cells were then harvested and western blotting was performed as described above.

### Seahorse assay

Oxygen consumption rate (OCR) and extracellular acidification rate (ECAR) were measured using the Seahorse biosciences XFe24 flux analyzer (Agilent Technologies, Santa Clara, CA, USA) according to the manufacturer’s instructions. Briefly, cells were seeded on collagen-coated seahorse XFe24 well plates at a density of 5 × 10^4^ cells/well overnight. Then, OCR was determined in basal assay medium followed by sequential treatment with 1 μM oligomycin A, 1 μM carbonyl cyanide 4-(trifluoromethoxy) phenylhydrazone (FCCP) and 1 μM rotenone/antimycin A. For the ECAR measurement, cells were seeded at a density of 5 × 10^4^ cells/well in glucose-free XF assay medium by the sequential addition of 10 mM glucose, 1 μM oligomycin, and 50 mM 2-deoxyglucose (2-DG). Measurements were normalized to the cell number in each well at the end of assay and analyzed using the Seahorse Wave software.

### Animal studies

All animal experiments were performed in accordance with Institutional Animal Care and Use Committee of Fourth Military Medical University in Xi’an, Shaanxi, China. For the CML tumor xenograft assay, 3 × 10^6^ virally transduced K562 cells were inoculated subcutaneously into the right flank of nude mice. Tumor volume was measured and calculated as indicated [50]. Mice were euthanized when the tumor volume reached 1000 mm^3^. For the engraftment of human cells in immunodeficient mice. The primary CML cells (1 × 10^6^ cells/mouse) were transplanted by tail vein injection into sublethally irradiated (300 cGy) 8-week-old NSI mice (Cyagen, Guangzhou, China). Mice were euthanized after 12 weeks, and BM cells and spleen cells were harvested; the engraftment of human cells was analyzed by flow cytometer. For the CML transformation model, MSCV-BCR-ABL-Cre-GFP and MSCV-BCR-ABL-TXNIP-GFP constructs were generated as previously described [26]. Retroviral transduction of BM cells and transplantation were performed [48]. Briefly, MSCV-BCR-ABL-Cre-GFP, MSCV-BCR-ABL-TXNIP-GFP, and MSCV-BCR-ABL-GFP vectors were packaged using Phoenix-Eco cells, and viral stocks were used for BM transduction. Donor mice (TXNIP^fl/fl^ or C57BL/6 mice) were primed by intraperitoneal injection with one dose of 200 mg/kg 5-fluorouracil 3 days before harvest. BM cells were harvested and cultured in prestimulation medium (DMEM,15% heat-inactivated FBS, 5% WEHI-3B–conditioned medium, 500 U/mL of IL-6, 5 U/mL of SCF, and 120 U/mL of recombinant murine IL-3). After 24 h, the cells were transduced with retroviral vectors by 2 rounds of co-sedimentation transduction. Transduced cells were transplanted by tail vein injection. At day 10 after transplantation, drugs were administrated continuously for 10 days. Imatinib was administrated by oral gavage with 75 mg/kg in the morning and 125 mg/kg in the afternoon.JQ1 was given by intraperitoneal injection at 50 mg/kg/d. The BM progenitor cells were isolated and cell colony formation assay were performed as previously indicated [48].

### GEO, GTEx, and TCGA patient data analysis

For the gene expression analysis according to the clinical public datasets, we obtained GSE47927 (patients with CML in accelerated (AP) phase:15, blastic (BC) phase:9), datasets from GEO database (https://www.ncbi.nlm.nih.gov/geo/). The TXNIP expression differences between AP phase and BC phase in these datasets were compared by Student’s t-test, which were visualized through “GEOquery” and “ggplot2” package in R software. *P* < 0.05 was regarded as statistically significant. The Gene Expression Profiling Interactive Analysis (GEPIA) was used to determine the correlation of c-Myc and TXNIP according to The Cancer Genome Atlas (TCGA) or Genotype-Tissue Expression (GTEx) dataset.

### Statistical analysis

Data are expressed as mean ± SD. Statistical analysis was performed with the SPSS26.0 software package by using student’s t-test for independent groups. Statistical significance was based on a value of *P* ≤ 0.05.

## Supplementary information

aj-checklist

Supplementary Figure Legend

Supplementary Figure 1

Supplementary Figure 2

Supplementary Figure 3

Supplementary Figure 4

Supplementary Table1–3

Original Data File

## Data Availability

The gene expression profile by microarray in this paper has been deposited in NCBI GEO: GSE218451.
