# Supplementary material for: BCR-ABL triggers a glucose-dependent survival program during leukemogenesis through the suppression of TXNIP
Source: Cell Death Dis. 2023 Apr 24;14(4):287. doi: 10.1038/s41419-023-05811-2 (PMC10125982; doi:10.1038/s41419-023-05811-2)
Supplement: Supplementary file 2 — Supplementary Figure Legend [file 41419_2023_5811_MOESM2_ESM.docx]

**Supplementary figure 1. TXNIP mRNA Expressions in CML cells.** (A) TXNIP mRNA levels were determined in K562 and KCL22 cells after TXNIP knockdown. (B) TXNIP mRNA levels were determined in K562, K562G and K562R cells.

**Supplementary figure 2. Schematic structures of triple gene expression and control vectors**. (A) The MSCV vector co-expressing BCR-ABL , Cre, and GFP . (B) The MSCV vector co-expressing BCR-ABL, TXNIP, and GFP.

**Supplementary figure 3. TXNIP knockdown promotes CML cell growth in nude mice.** (A) The tumor weight were measured and analyzed. (B-C) Immunohistochemistry staining (B) and statistical analysis (C) of TXNIP, c-Myc and Ki67 in the sections of tumor graft. *** P <0.001.

**Supplementary figure 4. Effects of JQ1 and imatinib treatment on physiological indexes.** Mice were subjected to the same 10 day treatment with imatinib, JQ1, or combination as described in Figure 7G. Body weight (A) was monitored during drug treatment, and total erythrocyte counts (B) were analyzed.
