# Supplementary figures and images for: BCR-ABL triggers a glucose-dependent survival program during leukemogenesis through the suppression of TXNIP
Source: Cell Death Dis. 2023 Apr 24;14(4):287. doi: 10.1038/s41419-023-05811-2 (PMC10125982; doi:10.1038/s41419-023-05811-2)

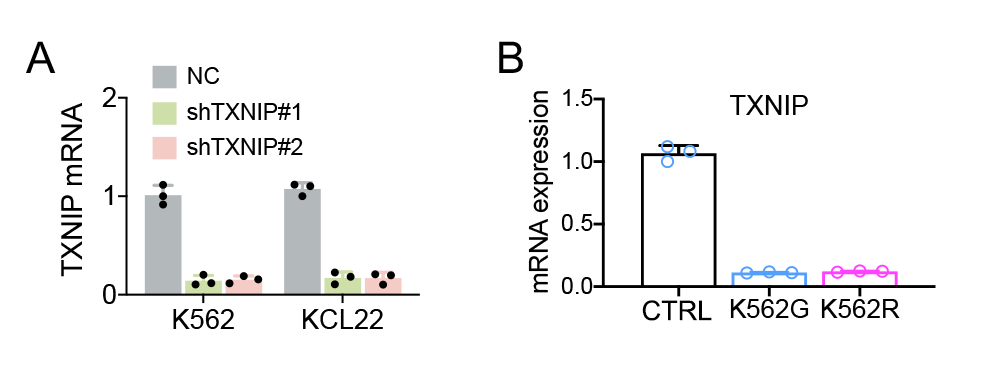

Supplement: Supplementary file 3 — Supplementary Figure 1 [file 41419_2023_5811_MOESM3_ESM.tif]

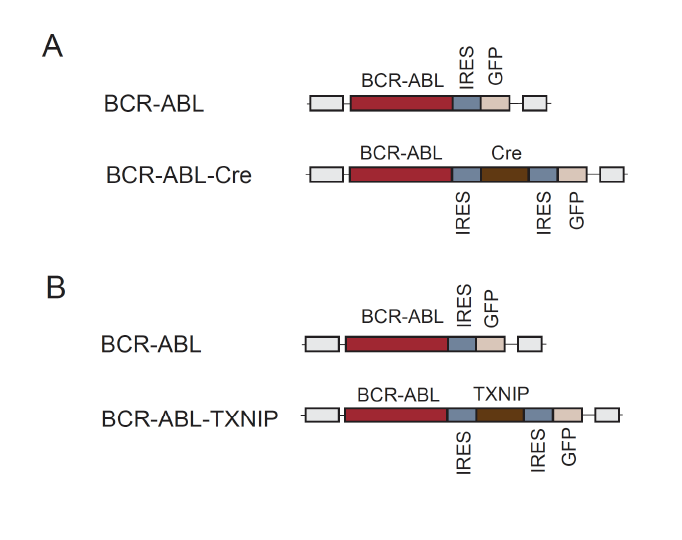

Supplement: Supplementary file 4 — Supplementary Figure 2 [file 41419_2023_5811_MOESM4_ESM.tif]

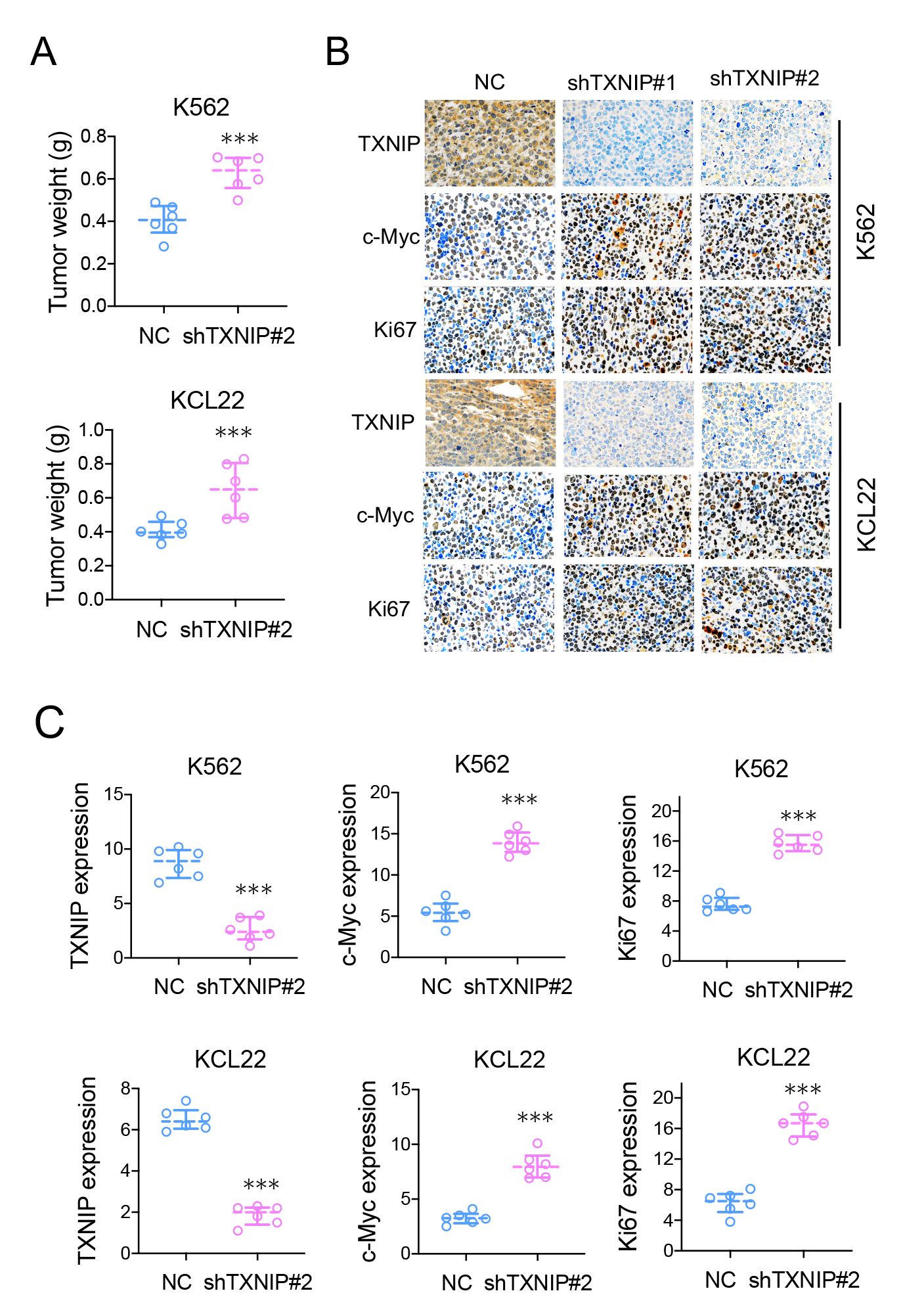

Supplement: Supplementary file 5 — Supplementary Figure 3 [file 41419_2023_5811_MOESM5_ESM.tif]

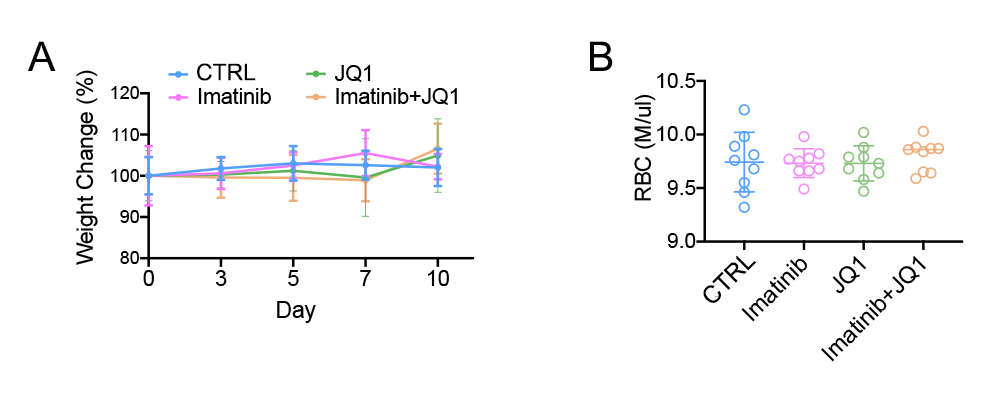

Supplement: Supplementary file 6 — Supplementary Figure 4 [file 41419_2023_5811_MOESM6_ESM.tif]
