# Supplementary material for: BCR-ABL triggers a glucose-dependent survival program during leukemogenesis through the suppression of TXNIP
Source: Cell Death Dis. 2023 Apr 24;14(4):287. doi: 10.1038/s41419-023-05811-2 (PMC10125982; doi:10.1038/s41419-023-05811-2)
Supplement: Supplementary file 8 — Original Data File [file 41419_2023_5811_MOESM8_ESM.pptx]

## Slide 1
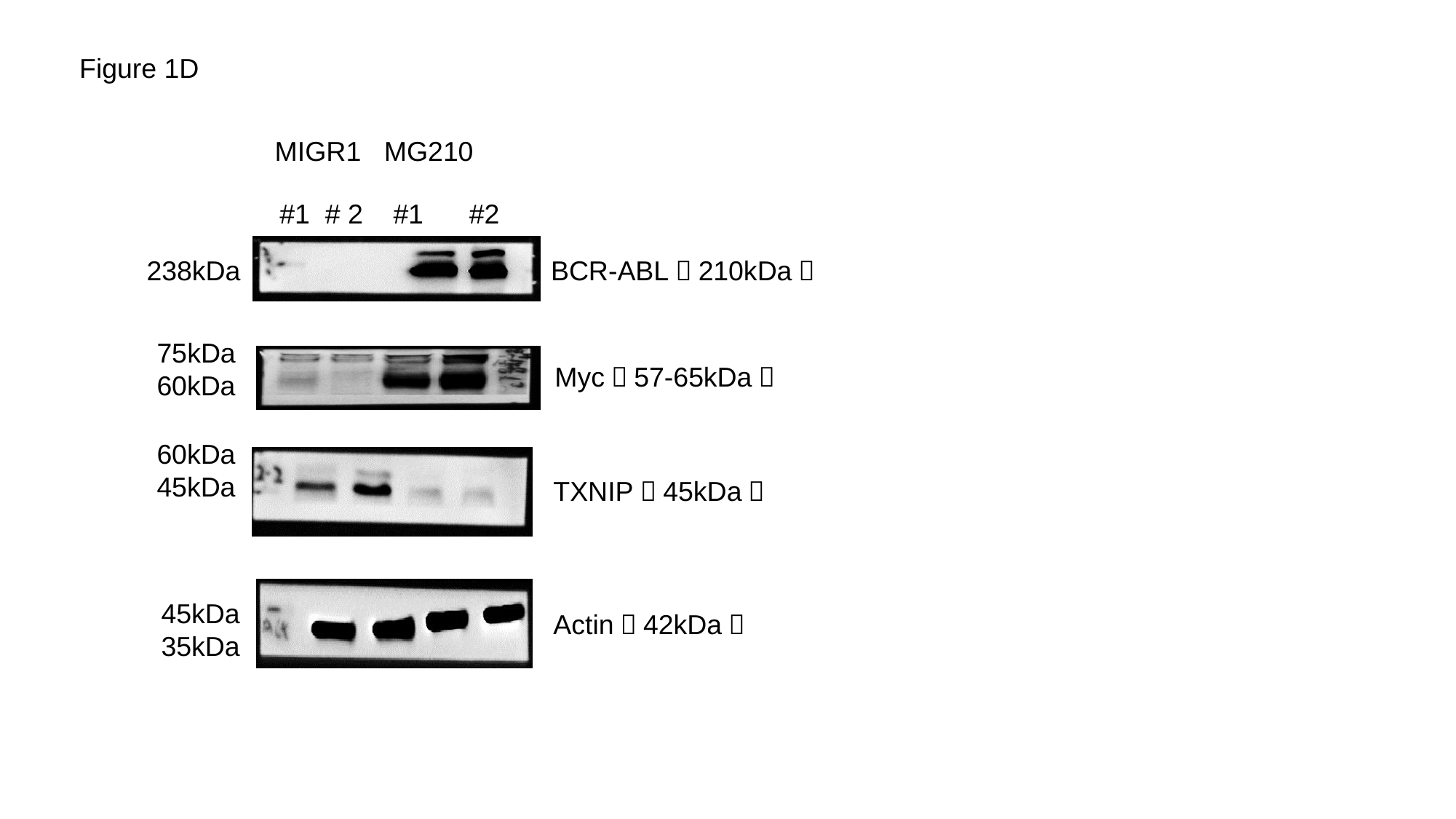

Figure 1D
MIGR1 MG210
 #1 # 2 #1 #2
238kDa
BCR-ABL（210kDa）
75kDa
60kDa
Myc（57-65kDa）
60kDa
45kDa
TXNIP（45kDa）
45kDa
35kDa
Actin（42kDa）

## Slide 2
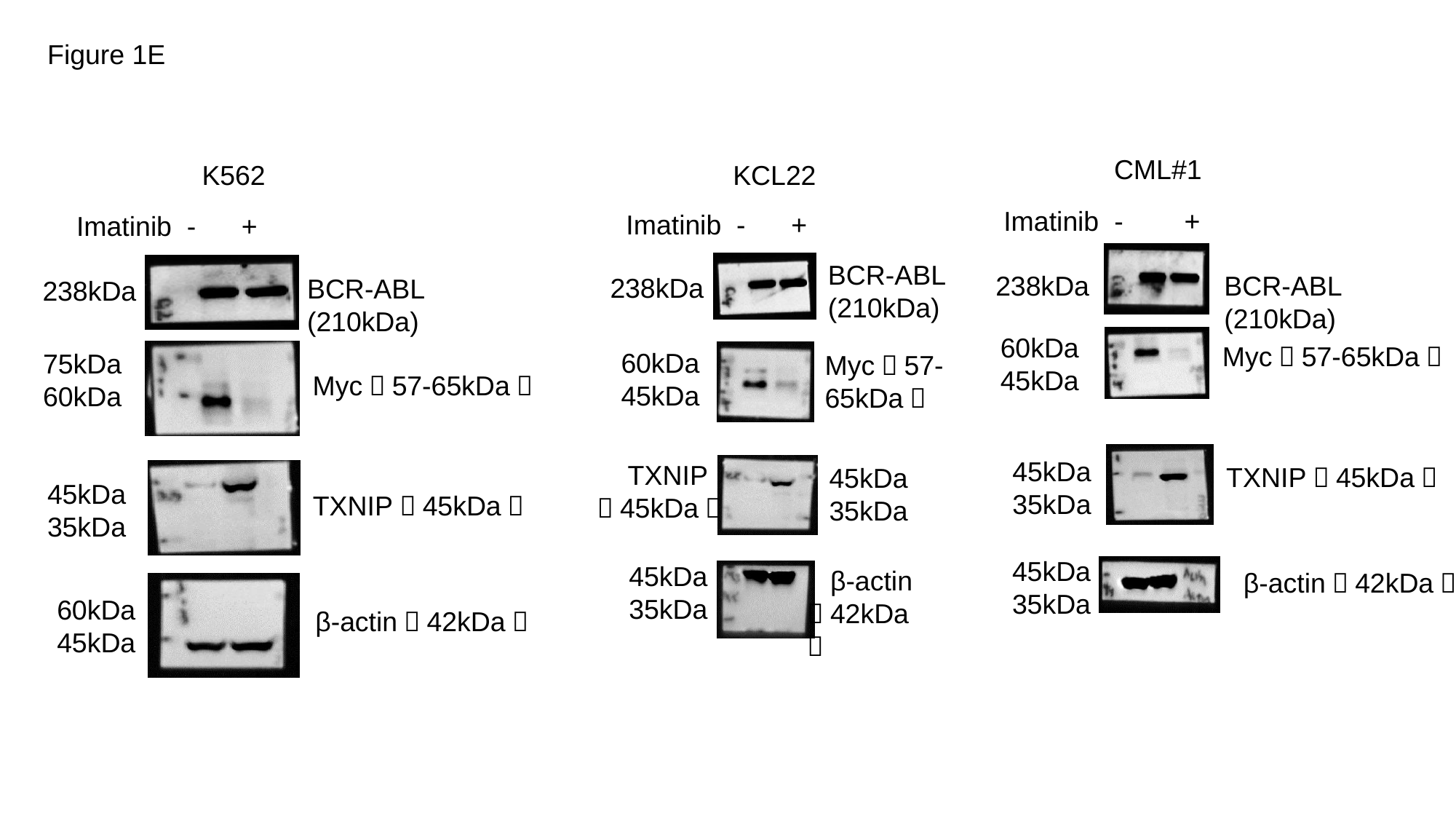

Figure 1E
CML#1
K562
KCL22
Imatinib - +
Imatinib - +
Imatinib - +
BCR-ABL
(210kDa)
238kDa
BCR-ABL (210kDa)
238kDa
BCR-ABL (210kDa)
238kDa
60kDa
45kDa
Myc（57-65kDa）
60kDa
45kDa
75kDa
60kDa
Myc（57-
65kDa）
Myc（57-65kDa）
45kDa
35kDa
 TXNIP
（45kDa）
TXNIP（45kDa）
45kDa
35kDa
45kDa
35kDa
TXNIP（45kDa）
45kDa
35kDa
45kDa
35kDa
 β-actin
（42kDa）
β-actin（42kDa）
60kDa
45kDa
β-actin（42kDa）

## Slide 3
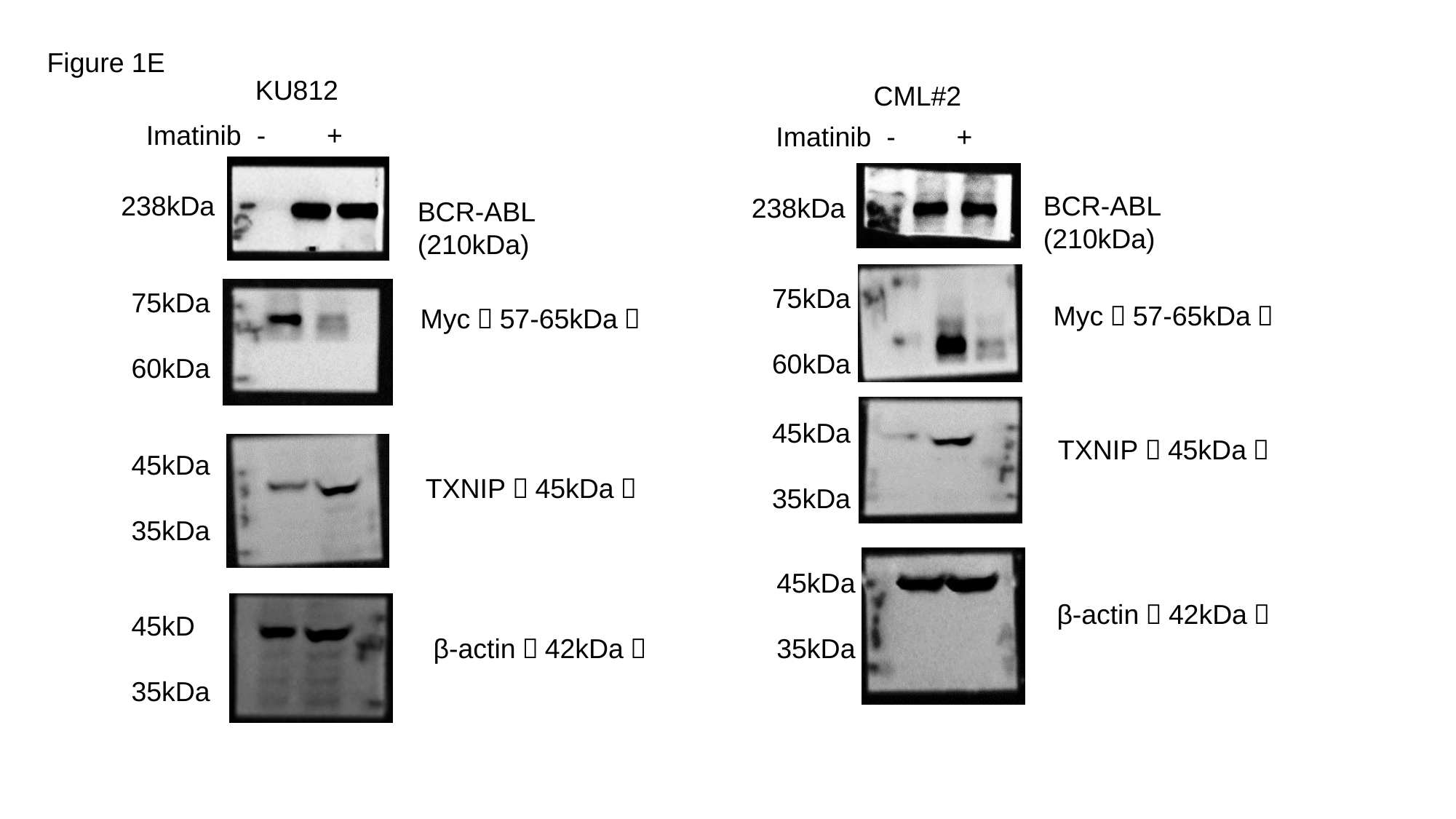

Figure 1E
KU812
CML#2
Imatinib - +
Imatinib - +
238kDa
BCR-ABL (210kDa)
238kDa
BCR-ABL (210kDa)
75kDa
60kDa
75kDa
60kDa
Myc（57-65kDa）
Myc（57-65kDa）
45kDa
35kDa
 TXNIP（45kDa）
45kDa
35kDa
TXNIP（45kDa）
45kDa
35kDa
β-actin（42kDa）
45kD
35kDa
β-actin（42kDa）

## Slide 4
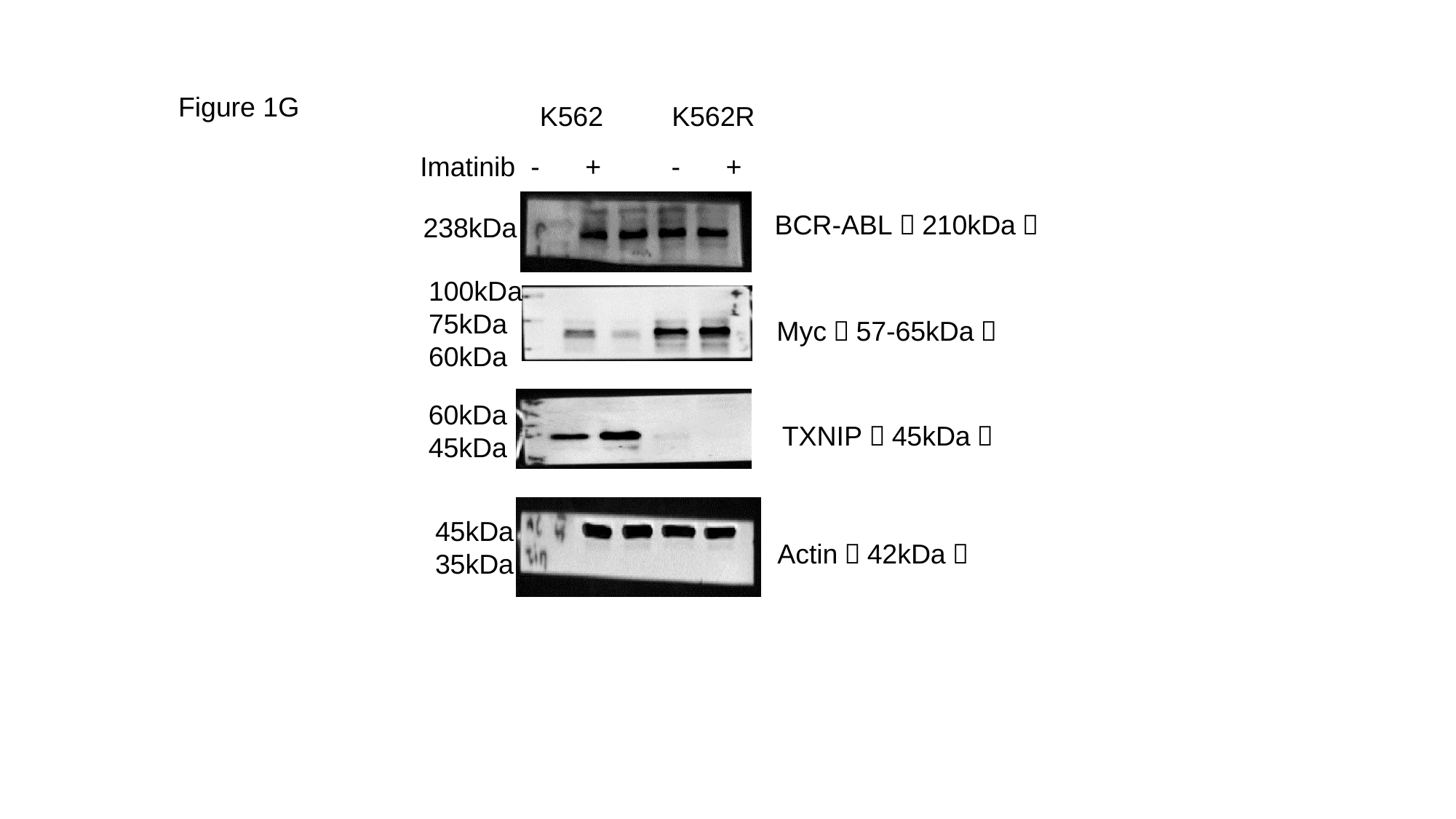

Figure 1G
K562
K562R
Imatinib - +
- +
BCR-ABL（210kDa）
238kDa
100kDa
75kDa
60kDa
Myc（57-65kDa）
60kDa
45kDa
TXNIP（45kDa）
45kDa
35kDa
Actin（42kDa）

## Slide 5
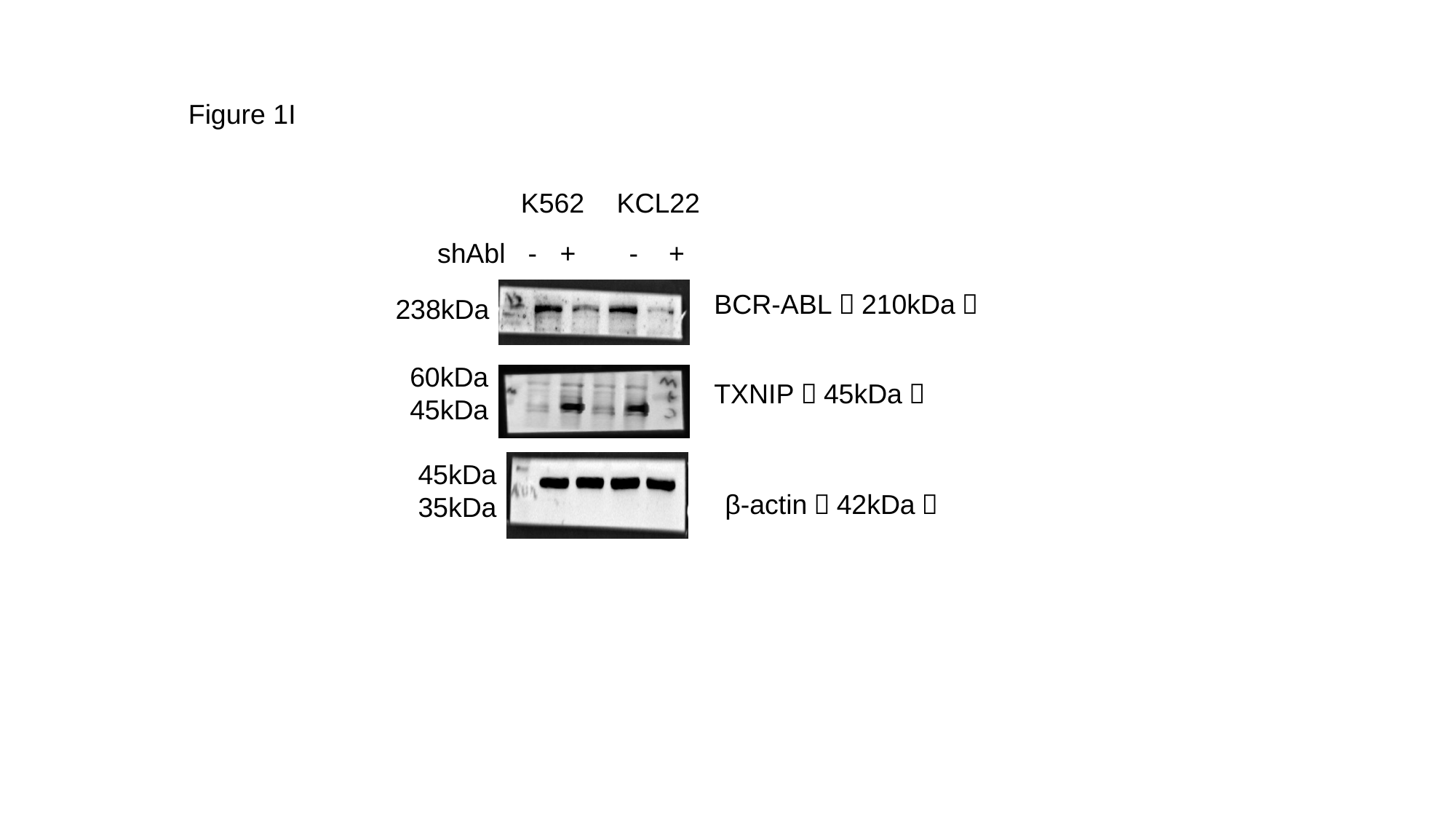

Figure 1I
K562
KCL22
shAbl - + - +
BCR-ABL（210kDa）
238kDa
60kDa
45kDa
 TXNIP（45kDa）
45kDa
35kDa
β-actin（42kDa）

## Slide 6
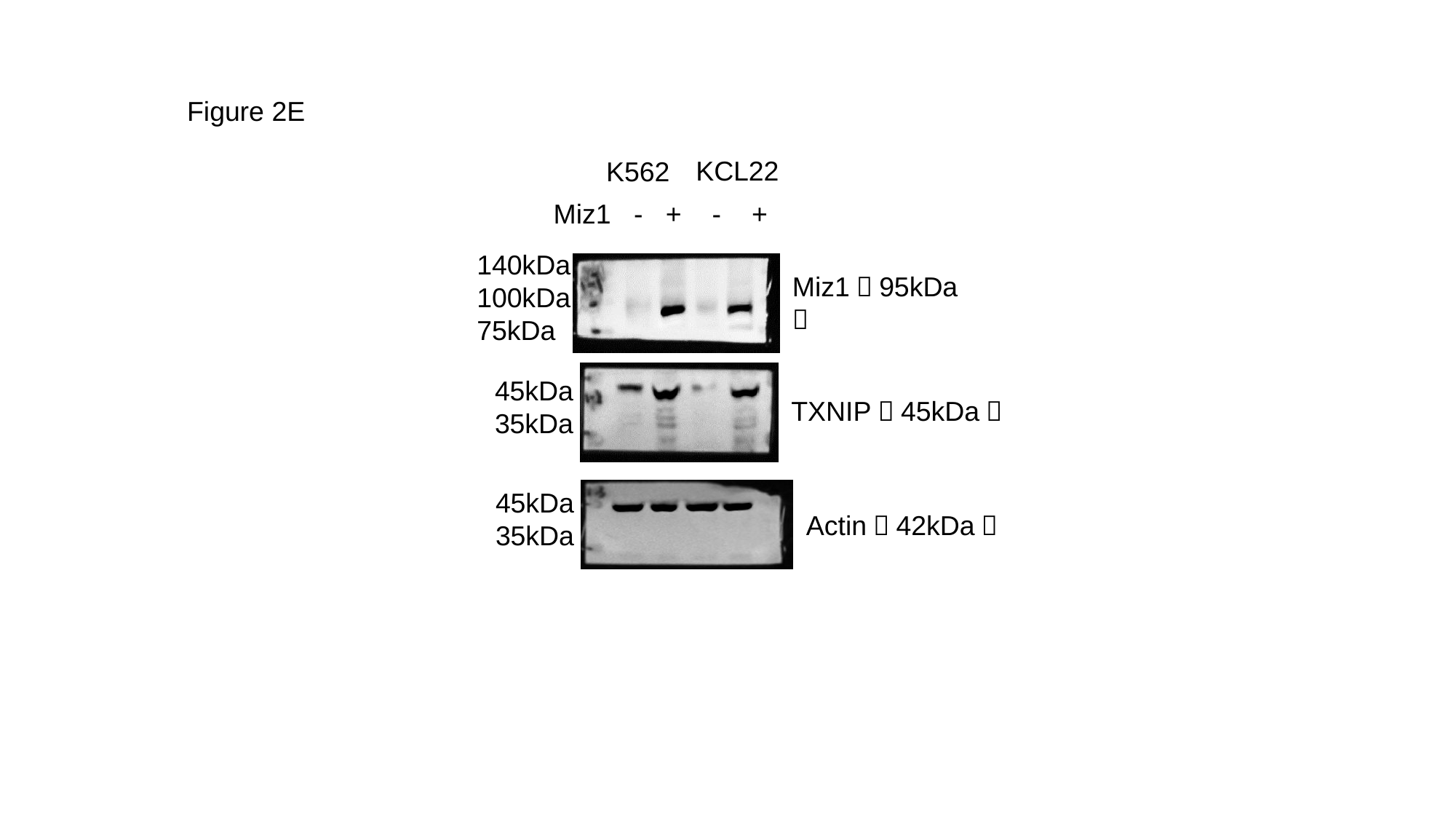

Figure 2E
KCL22
K562
Miz1 - + - +
140kDa
100kDa
75kDa
Miz1（95kDa）
45kDa
35kDa
TXNIP（45kDa）
45kDa
35kDa
Actin（42kDa）

## Slide 7
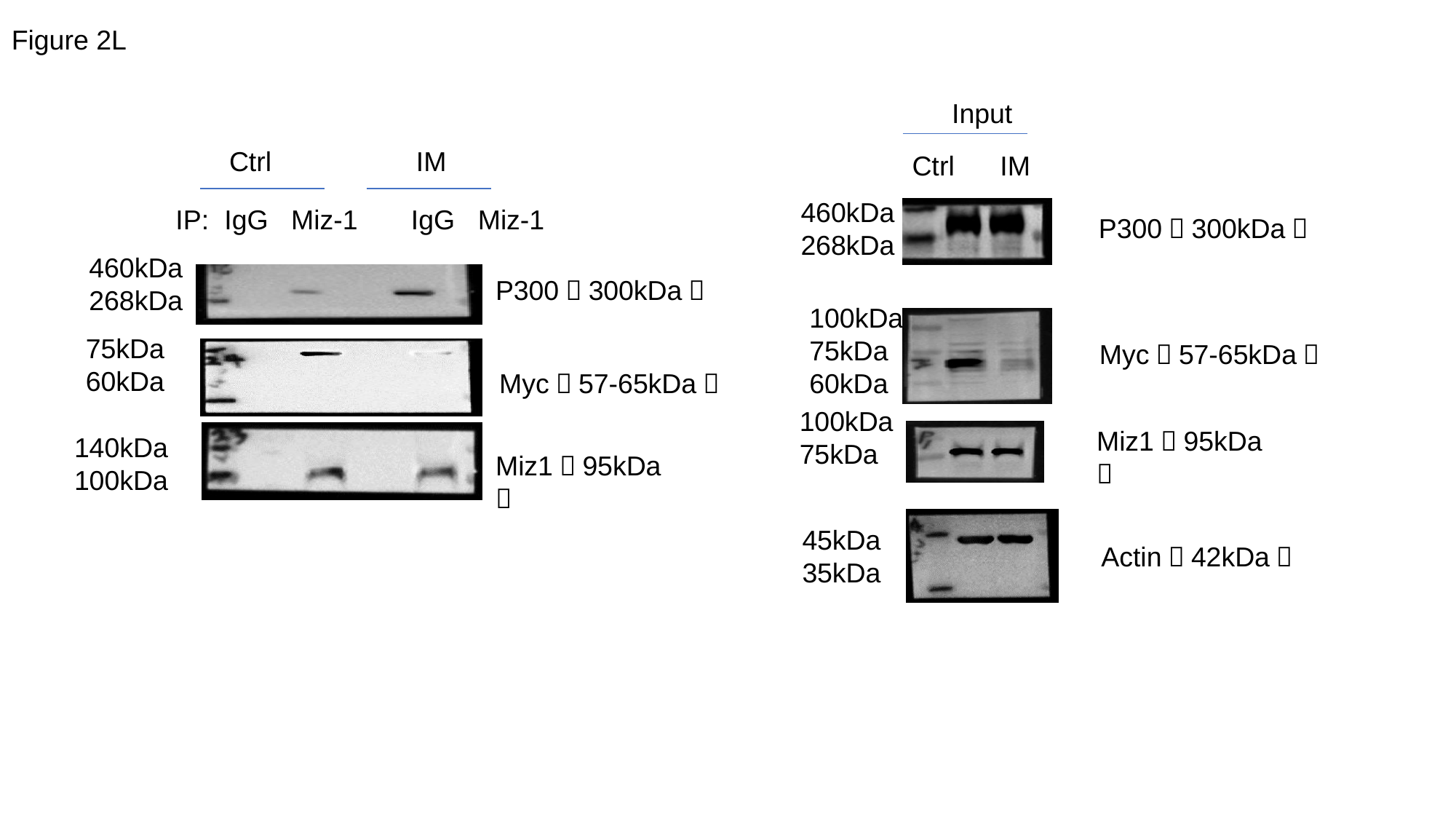

Figure 2L
Input
Ctrl IM
Ctrl IM
460kDa
268kDa
IP: IgG Miz-1 IgG Miz-1
P300（300kDa）
460kDa
268kDa
P300（300kDa）
100kDa
75kDa
60kDa
75kDa
60kDa
Myc（57-65kDa）
Myc（57-65kDa）
100kDa
75kDa
Miz1（95kDa）
140kDa
100kDa
Miz1（95kDa）
45kDa
35kDa
Actin（42kDa）

## Slide 8
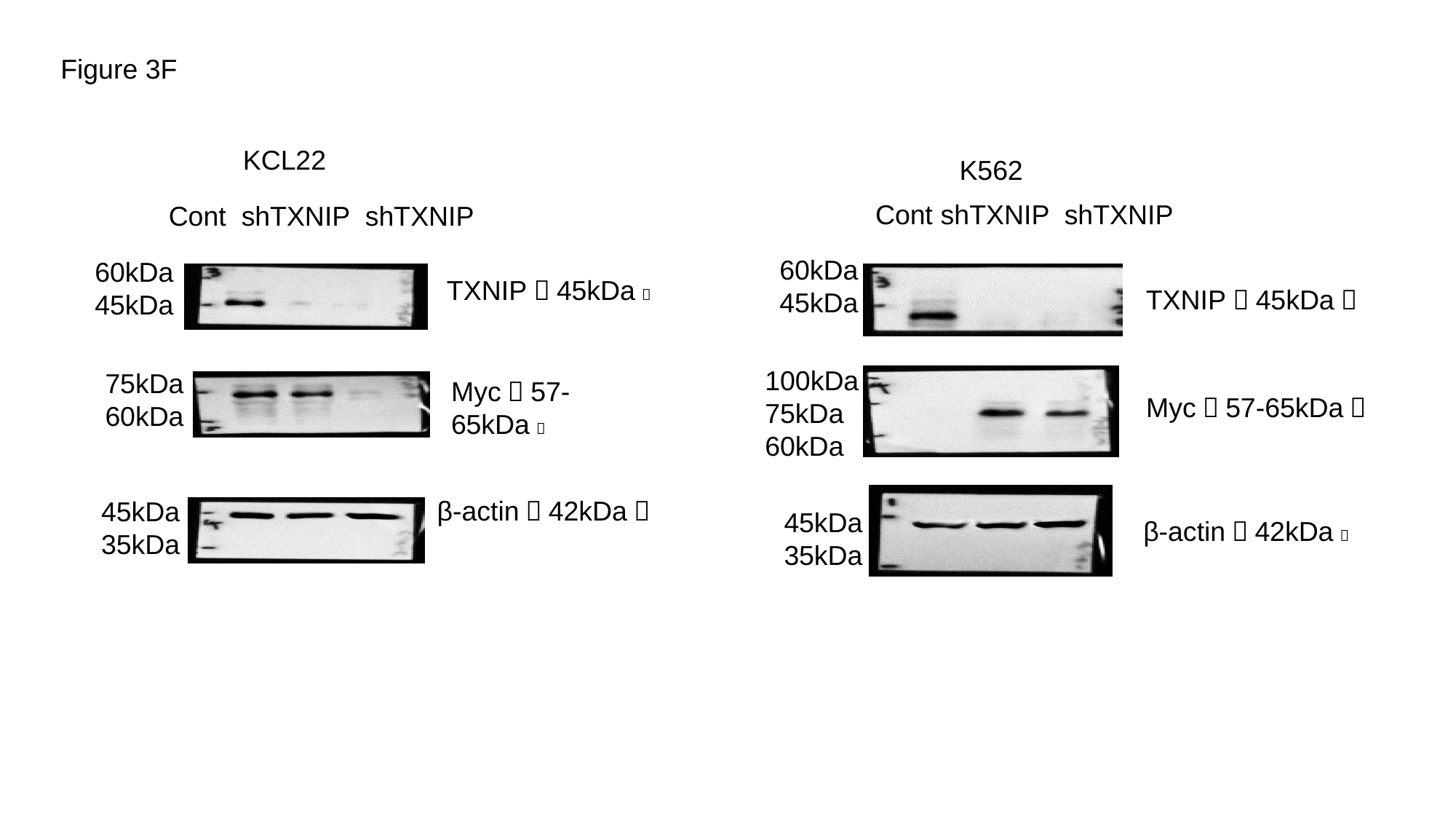

Figure 3F
KCL22
K562
Cont shTXNIP shTXNIP
Cont shTXNIP shTXNIP
60kDa
45kDa
60kDa
45kDa
TXNIP（45kDa）
TXNIP（45kDa）
100kDa
75kDa
60kDa
75kDa
60kDa
Myc（57-65kDa）
Myc（57-65kDa）
β-actin（42kDa）
45kDa
35kDa
45kDa
35kDa
β-actin（42kDa）

## Slide 9
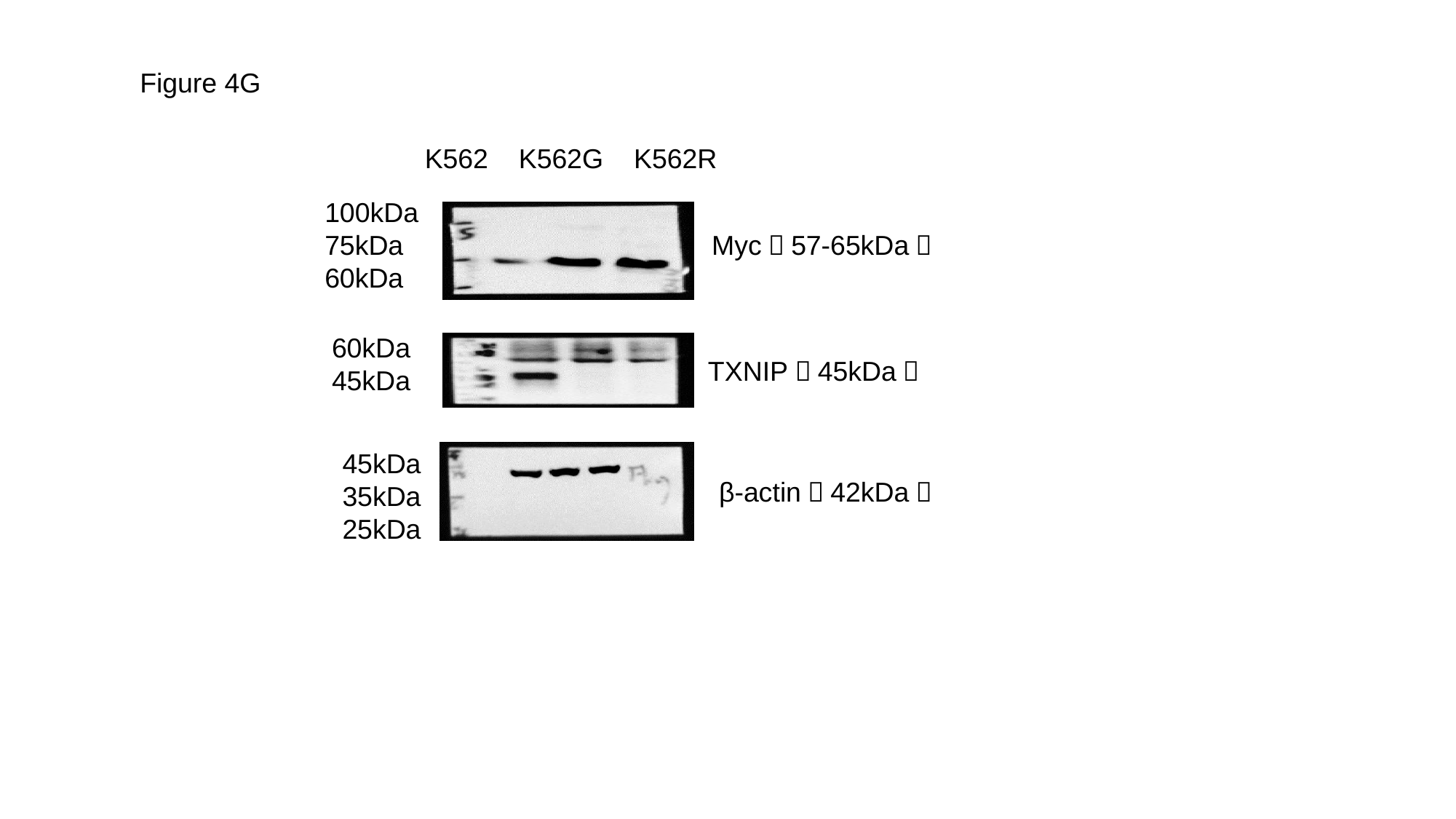

Figure 4G
K562 K562G K562R
100kDa
75kDa
60kDa
Myc（57-65kDa）
60kDa
45kDa
TXNIP（45kDa）
45kDa
35kDa
25kDa
β-actin（42kDa）

## Slide 10
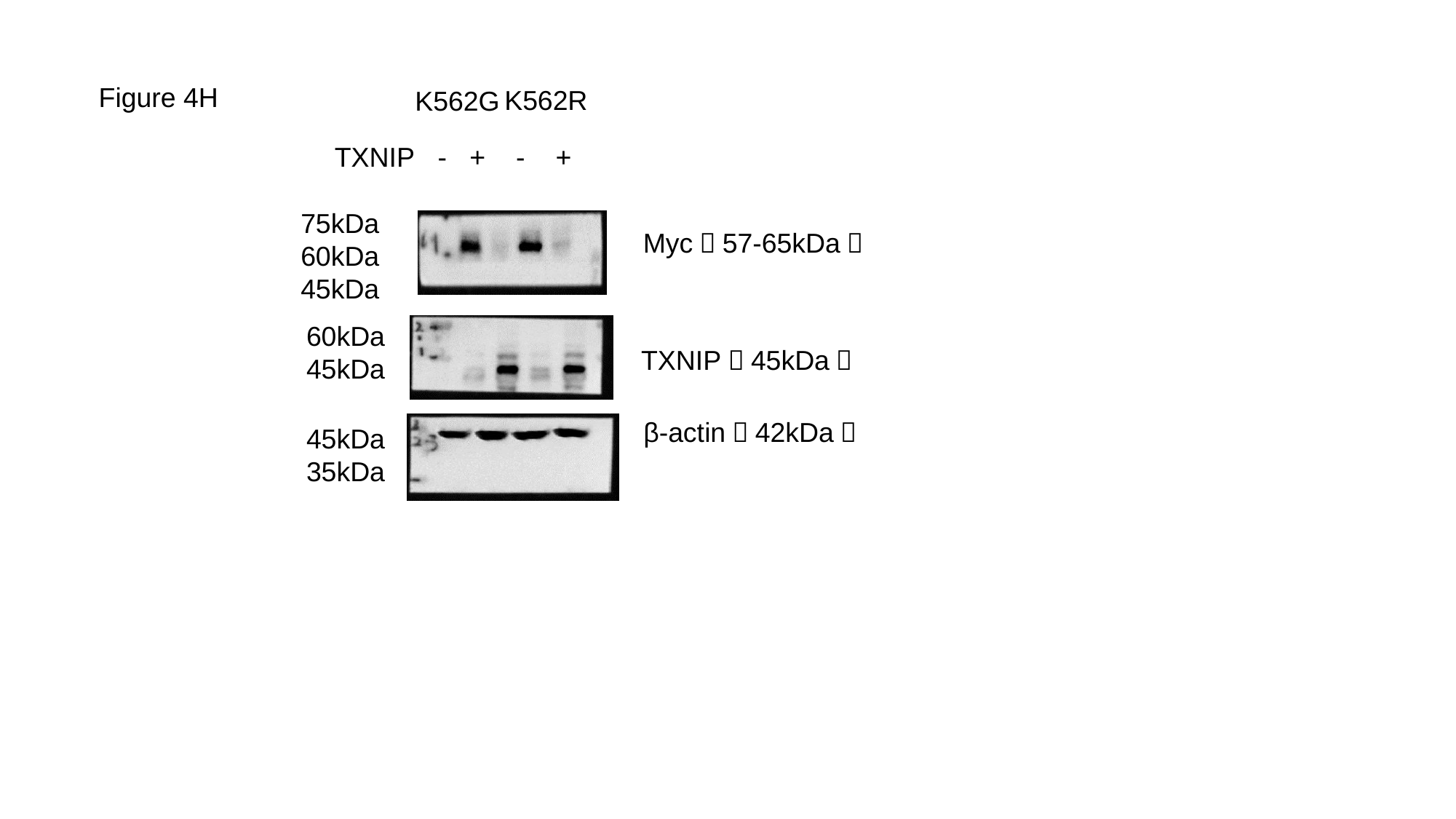

Figure 4H
K562R
K562G
TXNIP - + - +
75kDa
60kDa
45kDa
Myc（57-65kDa）
60kDa
45kDa
 TXNIP（45kDa）
β-actin（42kDa）
45kDa
35kDa

## Slide 11
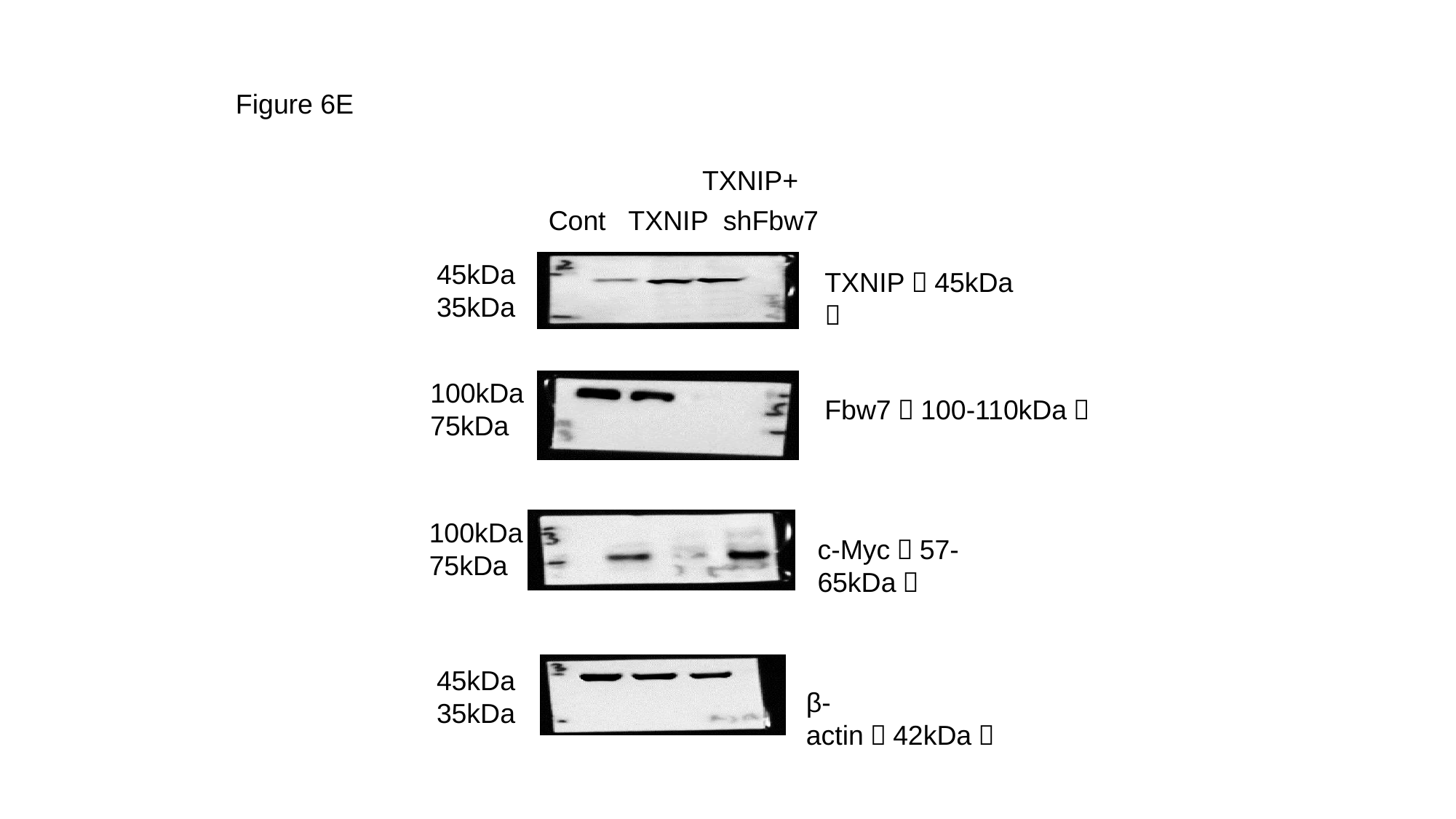

Figure 6E
TXNIP+
Cont TXNIP shFbw7
45kDa
35kDa
TXNIP（45kDa）
100kDa
75kDa
Fbw7（100-110kDa）
100kDa
75kDa
c-Myc（57-65kDa）
45kDa
35kDa
β-actin（42kDa）

## Slide 12
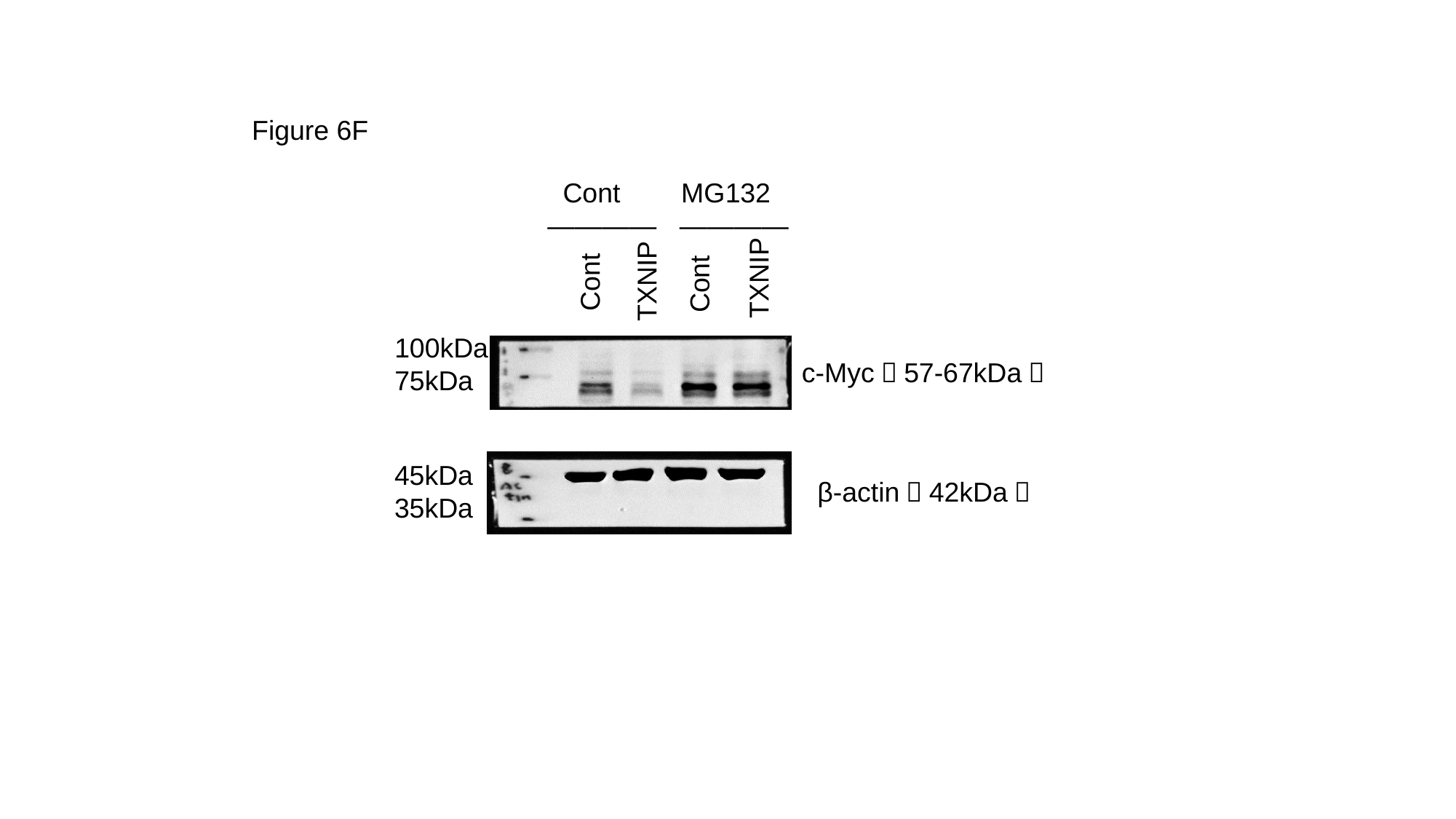

Figure 6F
 Cont MG132
―――― ――――
TXNIP
TXNIP
Cont
Cont
100kDa
75kDa
c-Myc（57-67kDa）
45kDa
35kDa
β-actin（42kDa）

## Slide 13
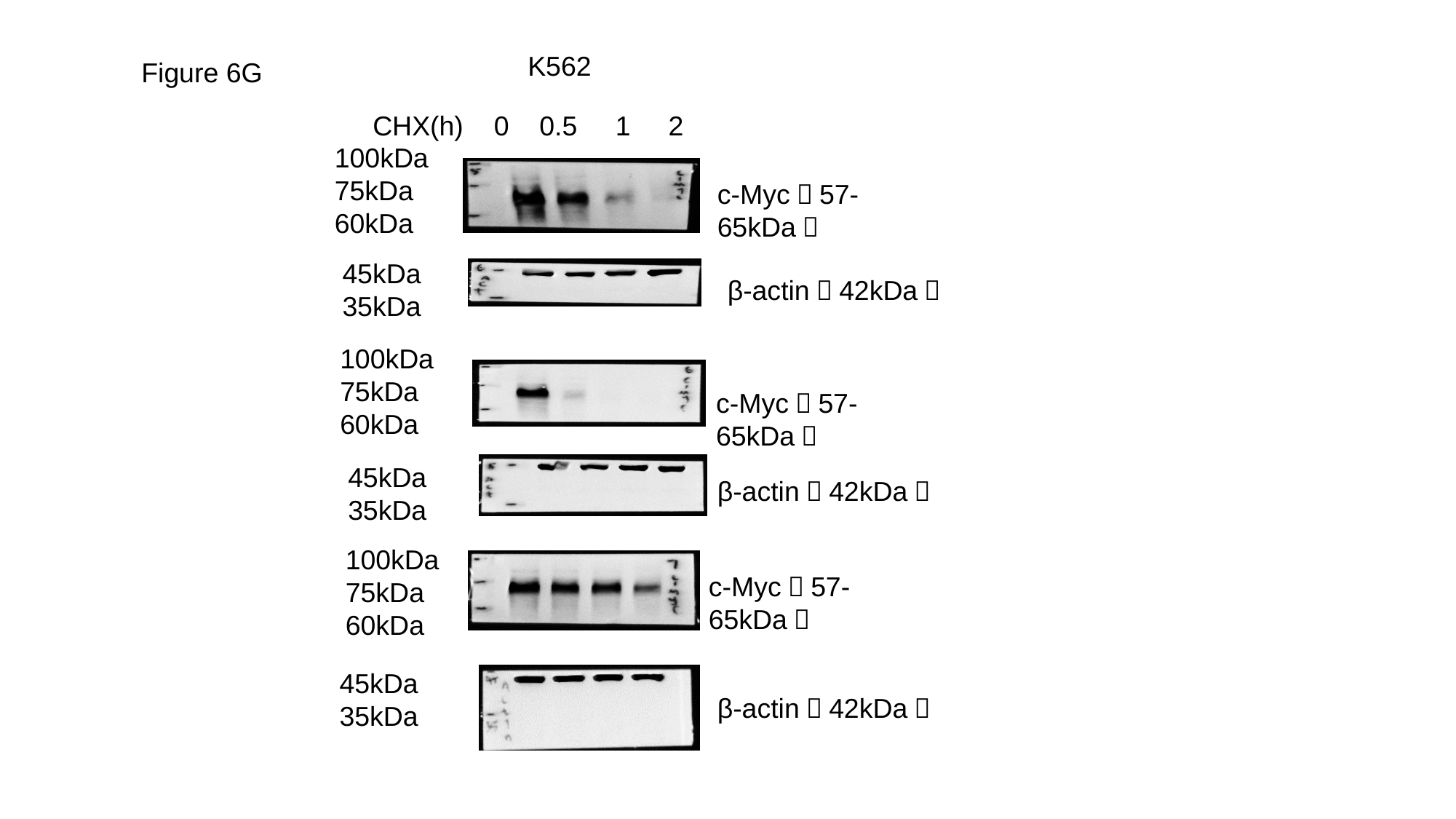

K562
Figure 6G
CHX(h) 0 0.5 1 2
100kDa
75kDa
60kDa
c-Myc（57-65kDa）
45kDa
35kDa
β-actin（42kDa）
100kDa
75kDa
60kDa
c-Myc（57-65kDa）
45kDa
35kDa
β-actin（42kDa）
100kDa
75kDa
60kDa
c-Myc（57-65kDa）
45kDa
35kDa
β-actin（42kDa）

## Slide 14
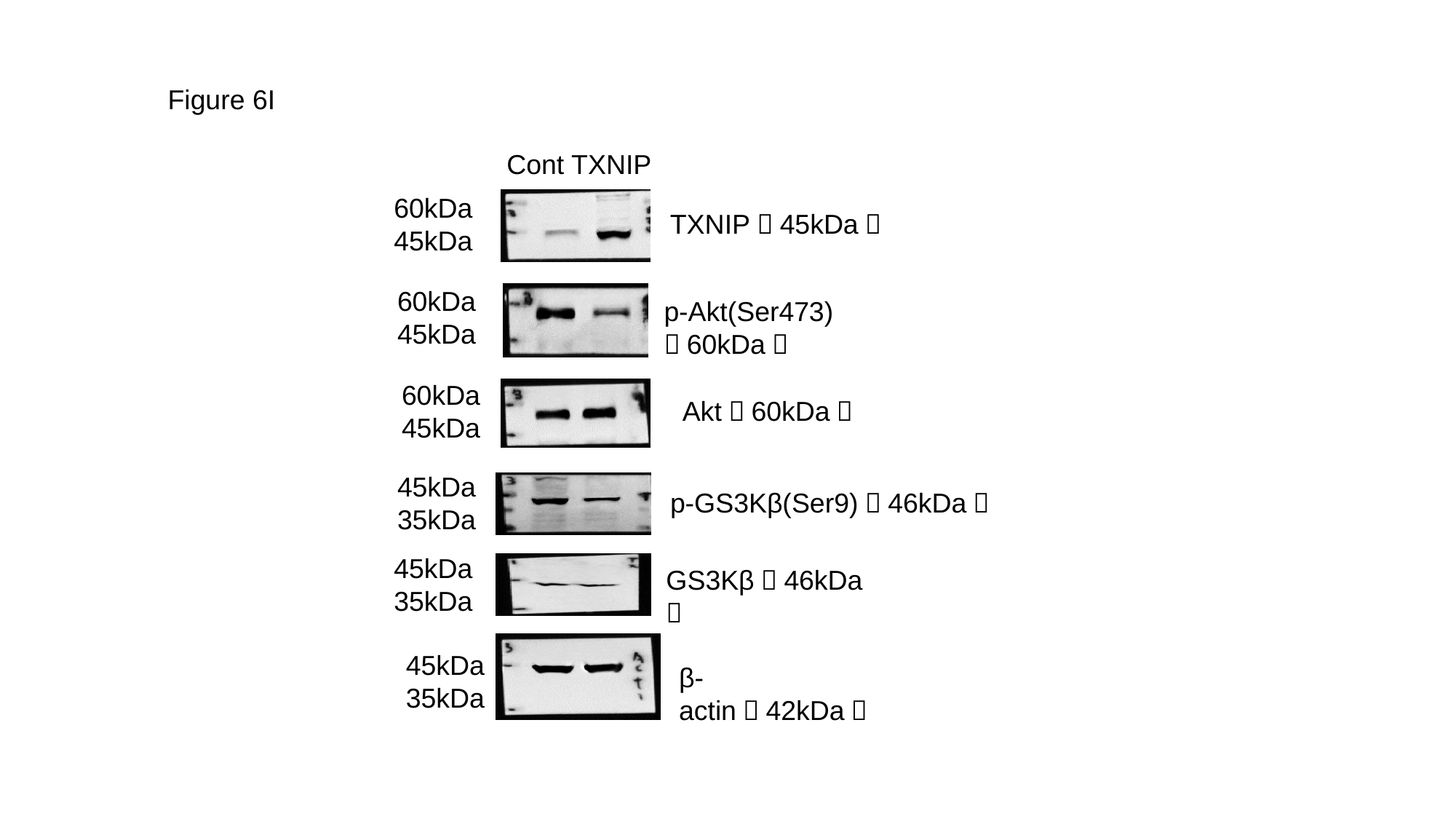

Figure 6I
Cont TXNIP
60kDa
45kDa
TXNIP（45kDa）
60kDa
45kDa
p-Akt(Ser473)（60kDa）
60kDa
45kDa
Akt（60kDa）
45kDa
35kDa
p-GS3Kβ(Ser9)（46kDa）
45kDa
35kDa
GS3Kβ（46kDa）
45kDa
35kDa
β-actin（42kDa）

## Slide 15
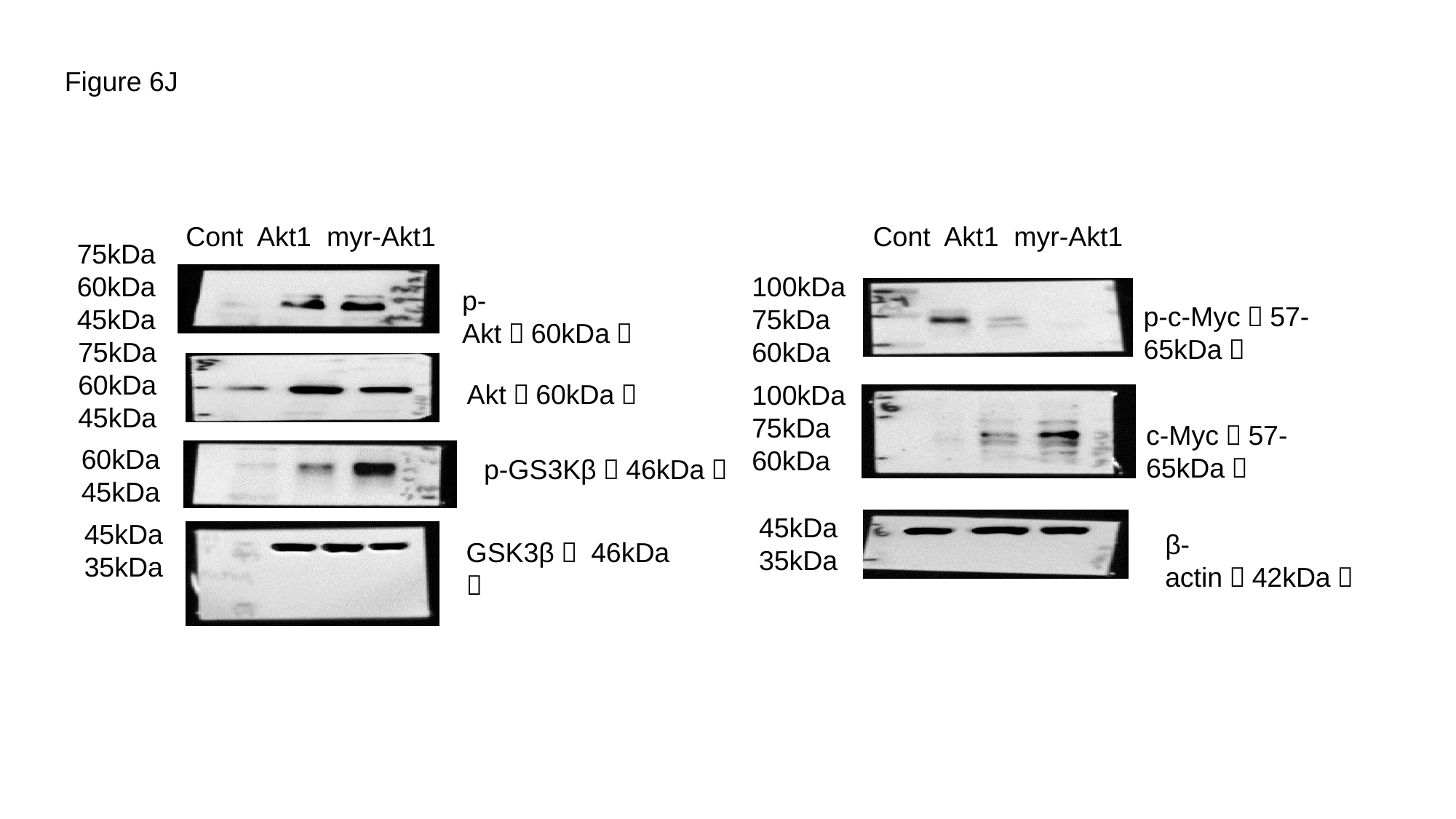

Figure 6J
Cont Akt1 myr-Akt1
Cont Akt1 myr-Akt1
75kDa
60kDa
45kDa
100kDa
75kDa
60kDa
p-Akt（60kDa）
p-c-Myc（57-65kDa）
75kDa
60kDa
45kDa
 Akt（60kDa）
100kDa
75kDa
60kDa
c-Myc（57-65kDa）
60kDa
45kDa
p-GS3Kβ（46kDa）
45kDa
35kDa
45kDa
35kDa
β-actin（42kDa）
GSK3β（ 46kDa ）

## Slide 16
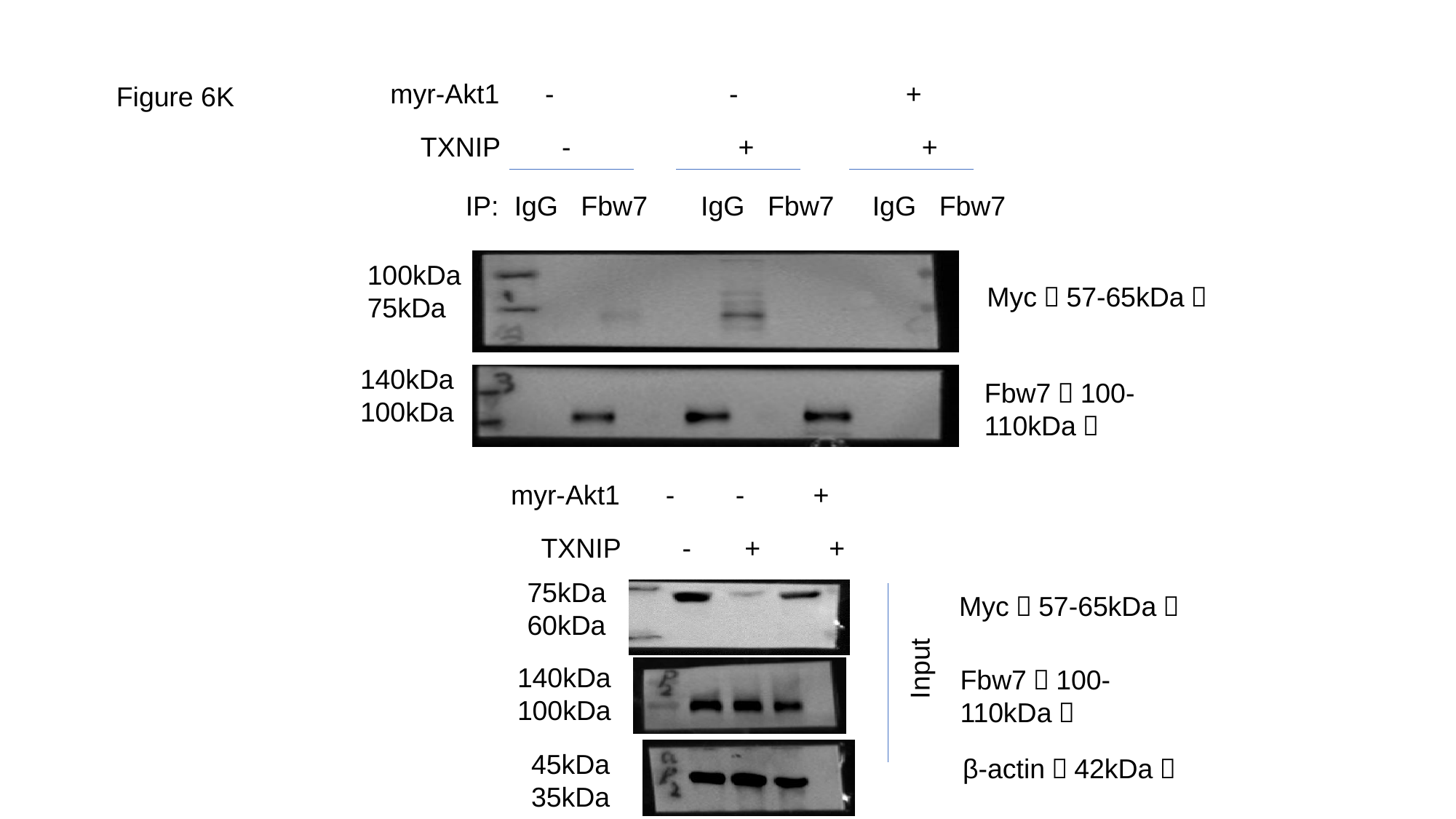

myr-Akt1 - - +
Figure 6K
TXNIP - + +
IP: IgG Fbw7 IgG Fbw7 IgG Fbw7
100kDa
75kDa
Myc（57-65kDa）
140kDa
100kDa
Fbw7（100-110kDa）
myr-Akt1 - - +
TXNIP - + +
75kDa
60kDa
Myc（57-65kDa）
Input
140kDa
100kDa
Fbw7（100-110kDa）
45kDa
35kDa
β-actin（42kDa）
